# ROS‐Responsive Eye Drop for Controlled Release of 20‐Deoxyingenol Improves Diabetic Corneal Healing by Activating Macrophage Autophagy and Metabolic Reprogramming

**DOI:** 10.1002/advs.77232

**Published:** 2026-08-18

**Authors:** Jiaxin Wu, Wanying Chen, Haijiang Qiu, Weihan Zheng, Ying Jiao, Jingheng Du, Zhudan Zhuang, Xing Hua, Jingyi Gu, Yuheng Liu, Zhaojin Li, Jingbin Xie, Hanxiao Sun, Huifei Lan, Xin Sun, Yunxia Xue, Ting Fu, Zhijie Li, Guobing Chen, Shiyu Li, Jun Liu

**Affiliations:** ^1^ International Ocular Surface Research Center Institute of Ophthalmology Key Laboratory for Regenerative Medicine Jinan University Guangzhou China; ^2^ Department of Ophthalmology The First Affiliated Hospital of Jinan University Jinan University Guangzhou China; ^3^ Department of Immunology & Institute of Geriatric Immunology School of Medicine Jinan University Guangzhou China; ^4^ Guangdong‐Hong Kong‐Macau Great Bay Area Geroscience Joint Laboratory School of Medicine Jinan University Guangzhou China; ^5^ Institute of Genomic Medicine College of Pharmacy Jinan University Guangzhou China; ^6^ Department of Ophthalmology Guangzhou First People's Hospital Guangzhou Guangdong China; ^7^ The First Affiliated Hospital of Jinan University Jinan University Guangzhou China; ^8^ Medical Experiment Research Center, School of Medicine Jinan University Guangzhou China

**Keywords:** 20‐deoxyingenol, autophagy, corneal wound healing, diabetes, inflammation, macrophage polarization, metabolic reprogramming, microspheres, smart‐responsive

## Abstract

Impaired corneal wound healing in diabetes remains a major clinical challenge, driven by defective autophagy, metabolic dysregulation, and sustained inflammation within a high‐reactive oxygen species (ROS) microenvironment. Here, we engineered a ROS‐responsive ocular delivery system based on N1‐(4‐boronobenzyl)‐N3‐(4‐boronophenyl)‐N1, N1, N3, N3‐tetramethylpropane‐1,3‐diaminium (TSPBA)/poly(vinyl alcohol) (PVA) hydrogel microspheres (TP hydrogel microspheres) encapsulating 20‐deoxyingenol (20‐DOI), which enables sustained and ROS‐triggered drug release. Under high‐glucose conditions in vitro, which were used to model a diabetic microenvironment associated with elevated oxidative stress, TP/20‐DOI microspheres promoted macrophage polarization from the M1 phenotype toward the M2 phenotype. Transcriptomic and metabolic analyses indicated that this shift was driven by enhanced autophagy and concurrent metabolic rewiring from glycolysis to oxidative phosphorylation. In streptozotocin‐induced diabetic mice with corneal abrasions, topical TP/20‐DOI microspheres significantly accelerated re‐epithelialization and nerve regeneration while attenuating inflammation. The mechanism involved the autophagy‐dependent suppression of macrophage M1 polarization, which diminished the levels of macrophage‐derived C‐X‐C motif chemokine ligand 3 (CXCL3)/CXCL5 and neutrophil infiltration, as evidenced by the reversal of these effects upon pharmacological autophagy inhibition. Our findings establish an “autophagy–metabolism–polarization” axis as the core mechanism through which the microenvironment‐responsive TP/20‐DOI microspheres coordinate diabetic corneal repair, providing a blueprint for immunometabolic reprogramming therapies in ROS‐driven diabetic complications.

## Graphical abstract

Schematic illustration of ROS‐responsive TSPBA‐PVA microspheres loaded with 20‐deoxyingenol (TP/20‐DOI) for diabetic corneal wound healing: (a) fabrication and ROS‐triggered release; (b) autophagy activation and metabolic reprogramming in macrophages; (c) suppression of macrophage‐derived CXCL3/CXCL5, attenuation of neutrophil infiltration, and enhancement of corneal repair under diabetic conditions.

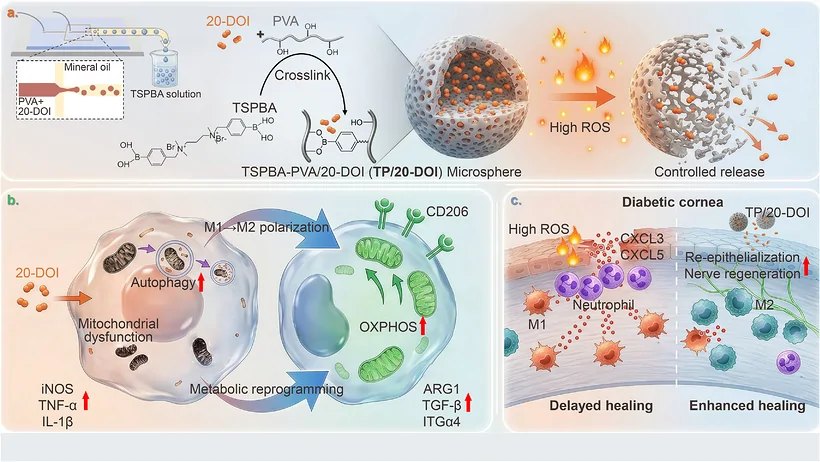

## 1 Introduction

The rapidly rising prevalence of diabetes has made ocular complications a major global public health threat to vision [1, 2, 3, 4]. Diabetic keratopathy, a common yet frequently overlooked ocular complication, manifests as various structural and functional abnormalities of the cornea. These include tear film system impairment [5, 6], corneal neuropathy [7], reduced corneal sensitivity [8], corneal edema [9], epithelial basement membrane abnormalities [5], and delayed corneal epithelial wound healing [10, 11]. Persistent corneal epithelial defects predispose the tissue to microbial infection, potentially progressing to ulceration, scarring, or opacity, ultimately threatening vision [3, 7, 12]. Studies have indicated that impaired autophagy [13, 14], an imbalance in macrophage polarization (characterized by sustained pro‐inflammatory M1 activation), and excessive neutrophil infiltration [15, 16, 17] are the central mechanisms hindering corneal wound healing. Although research on therapies for delayed diabetic corneal repair has advanced, effective treatments enabling rapid and complete healing are still lacking [3, 18]. Consequently, developing targeted strategies that simultaneously address dysregulated corneal immune cells (macrophages and neutrophils) and defective autophagy represents a critical therapeutic imperative.

In this study, we focused on the autophagy activator 20‐Deoxyingenol (20‐DOI), a diterpenoid compound derived from the roots of *Euphorbia kansui*. 20‐DOI exhibits beneficial effects in multiple disease models through its dual role in activating autophagy and suppressing inflammation. Specifically, it activates autophagy to inhibit oxidative stress‐induced chondrocyte apoptosis and senescence, thereby delaying osteoarthritis progression in mice [19]. Concurrently, it exerts anti‐inflammatory effects by suppressing the cyclic GMP‐AMP synthase (cGAS)‐stimulator of interferon genes (STING) signaling pathway, which in turn counteracts nucleus pulposus cell senescence and decelerates intervertebral disc degeneration in vitro and in vivo [20]. Further supporting its anti‐inflammatory property, 20‐DOI significantly inhibits nitric oxide production in vitro [21]. Moreover, it demonstrates neuroprotective activity by protecting hippocampal neurons from morphine‐induced neurotoxicity and improving spatial memory in rats [22].

Topical eye drops are the most common drug delivery method for anterior segment diseases but suffer from several inherent limitations [23]. First, low bioavailability (typically less than 5%) results in insufficient drug concentrations at the ocular target site, limiting therapeutic efficacy [23, 24]. Second, short ocular surface residence time—tear film clearance mechanisms rapidly remove instilled drugs, preventing sustained therapeutic action [25, 26]. Third, physiological barriers limit permeation; the complex ocular surface barriers (e.g., the multi‐layered cornea and tear film) hinder drug penetration, particularly for large molecules, reducing drug accessibility [27, 28, 29]. These limitations stem from the eye's sophisticated defense systems, comprising both static barriers and dynamic clearance mechanisms, which pose significant challenges for developing efficient delivery systems. Elevated reactive oxygen species (ROS) on the diabetic ocular surface [30, 31] provide a biochemical rationale for developing responsive biomaterial carriers, which can promote sustained drug release and improve bioavailability. However, creating clinically viable delivery technologies that reliably overcome ocular surface barriers remains an unmet need.

Hydrogels, three‐dimensional networks formed by crosslinked natural or synthetic polymers, are widely used in various disease models, including ophthalmic research [32, 33]. TSPBA‐PVA (TP) hydrogel is formed via crosslinking of N1‐(4‐boronobenzyl)‐N3‐(4‐boronophenyl)‐N1, N1, N3, N3‐tetramethylpropane‐1,3‐diaminium (TSPBA) and poly(vinyl alcohol) (PVA). Studies show that the phenylboronic acid groups in TSPBA and hydrogen bonds in PVA act synergistically to achieve robust and stable adhesion to skin, reducing the need for frequent administration [34]. PVA within the polymer can enhance drug permeability across the porcine cornea in vitro [35]. Furthermore, under high ROS concentrations, the boronic acid groups are oxidized, leading to hydrolysis or cleavage of the boronic ester bonds between TSPBA and PVA, causing hydrogel network disintegration and the release of encapsulated drugs. This property enables the hydrogel to scavenge ROS while responsively releasing drugs [36, 37]. These properties position TP as a promising platform for ocular surface drug delivery in ROS‐elevated pathological settings.

Theoretically, diabetic corneal repair can be framed by a hierarchical “autophagy‐immunometabolism‐polarization” cascade. Autophagy functions as a core cellular homeostatic program that maintains organelle integrity and mitochondrial quality control [38, 39]; by shaping mitochondrial fitness, it can bias cellular bioenergetics and drive immunometabolic state transitions (e.g., glycolysis dominant vs. oxidative phosphorylation dominant metabolism). In macrophages, such immunometabolic rewiring is tightly coupled to functional polarization outputs—pro inflammatory M1 programs vs. reparative M2 programs—which subsequently dictate the inflammatory microenvironment and leukocyte recruitment [15, 16, 17]. A key translational gap is that, despite this mechanistic logic, conventional ocular eye drop delivery cannot reliably sustain an upstream trigger sufficient to engage this cascade in a spatiotemporally controlled manner because of poor bioavailability and rapid clearance [23, 24, 25, 26]. In this context, the ROS rich diabetic corneal microenvironment [29, 30] represents an endogenous, disease encoded trigger that can be exploited to materialize upstream control of the axis, while TP based microspheres offer a strategy to couple trigger‐dose‐spatiotemporal control to overcome ocular surface barriers and enable sustained activation where and when needed [36, 37].

Based on the above challenges and rationale, we propose an innovative therapeutic strategy: constructing a ROS‐responsive, ocular‐surface‐deliverable TP hydrogel microsphere for encapsulating and targeted delivery of 20‐DOI to modulate the diabetic corneal wound microenvironment and promote healing. The core design lies in leveraging abnormally elevated ROS levels at the diabetic corneal wound site to trigger microsphere degradation and controlled drug release, thereby overcoming key limitations of traditional eye drops (low bioavailability, short residence time, and poor permeability) [23, 24, 25, 26, 27, 28, 29]. We hypothesize that TP/20‐DOI microspheres, through ROS‐responsive release of 20‐DOI, initiate macrophage autophagy, drive metabolic reprogramming (from glycolysis to oxidative phosphorylation), and thereby promote polarization from a pro inflammatory M1 phenotype to a reparative M2 phenotype; in parallel, in an autophagy dependent manner, they suppress macrophage derived neutrophil chemokines C‐X‐C motif chemokine ligand 3 (CXCL3)/CXCL5, thereby reducing excessive neutrophil infiltration and ultimately synergistically promoting epithelial regeneration and nerve repair in the diabetic cornea (Scheme 1). Collectively, this work aims to provide not only a novel topical therapeutic approach for diabetic keratopathy but also mechanistic evidence supporting a scalable “autophagy‐metabolism‐polarization” framework for microenvironment responsive immunomodulation in tissue repair.

**SCHEME 1:**
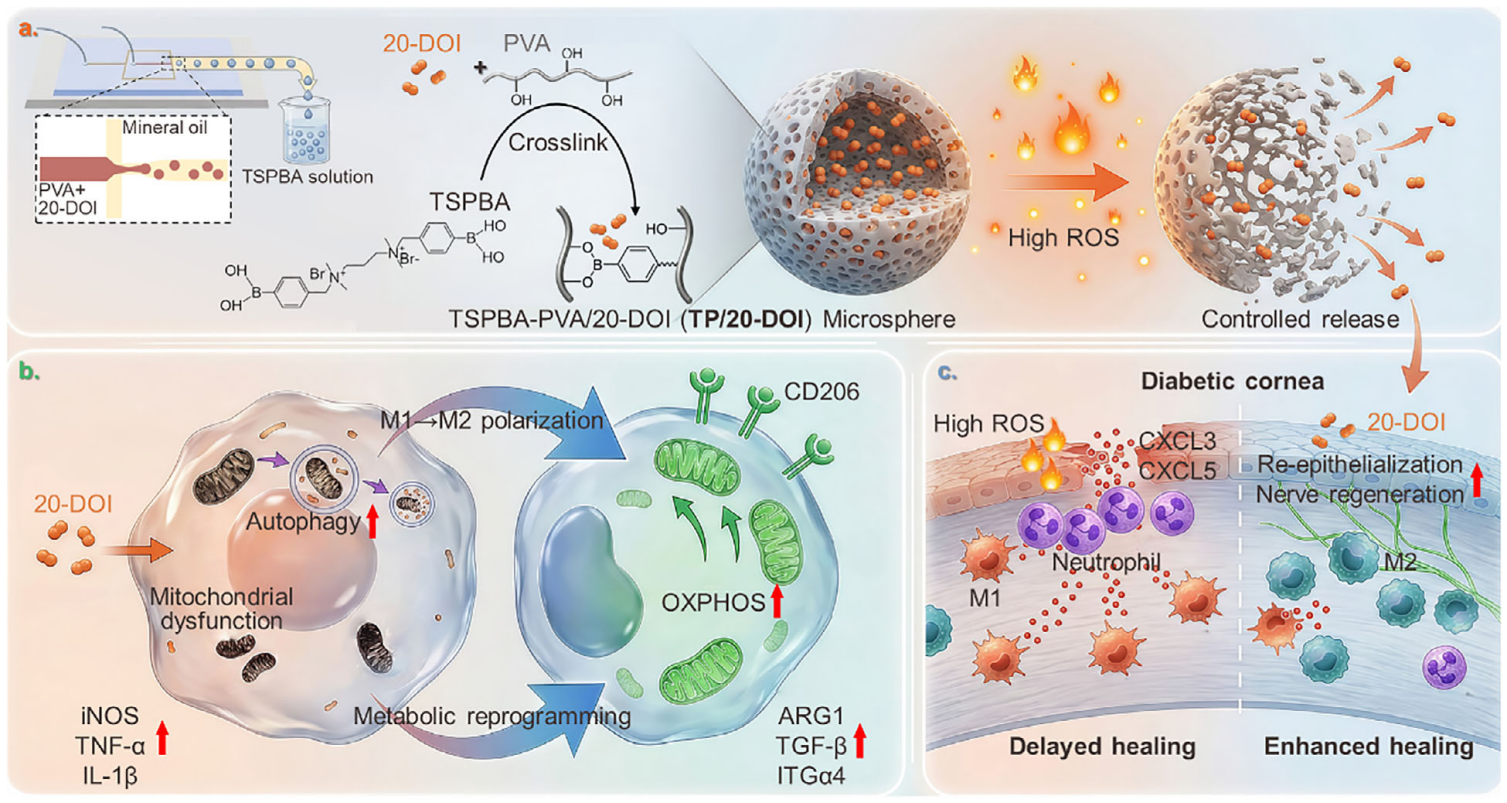
Schematic illustration of the fabrication and therapeutic mechanism of ROS‐responsive TSPBA‐PVA microspheres loaded with 20‐deoxyingenol (TP/20‐DOI) for diabetic corneal wound healing. (a) Preparation of TP/20‐DOI microspheres and ROS‐triggered drug release. (b) 20‐DOI promotes autophagy activation and metabolic reprogramming in macrophages. (c) ROS‐responsive release of 20‐DOI suppresses macrophage‐derived CXCL3 and CXCL5 secretion, thereby reducing neutrophil infiltration and promoting corneal wound healing under diabetic conditions.

## 2 Results

### 2.1 Preparation of TP/20‐DOI Microspheres

In diabetic conditions, sustained hyperglycemia markedly increases tissue ROS levels. 2′,7′‐dichlorodihydrofluorescein diacetate (DCFH‐DA) probe detection revealed significantly elevated ROS levels in the corneas of streptozotocin (STZ)‐induced diabetic mice following injury compared with the control group (Figure 1a), supporting the use of ROS as an endogenous trigger for ocular drug release.

**FIGURE 1:**
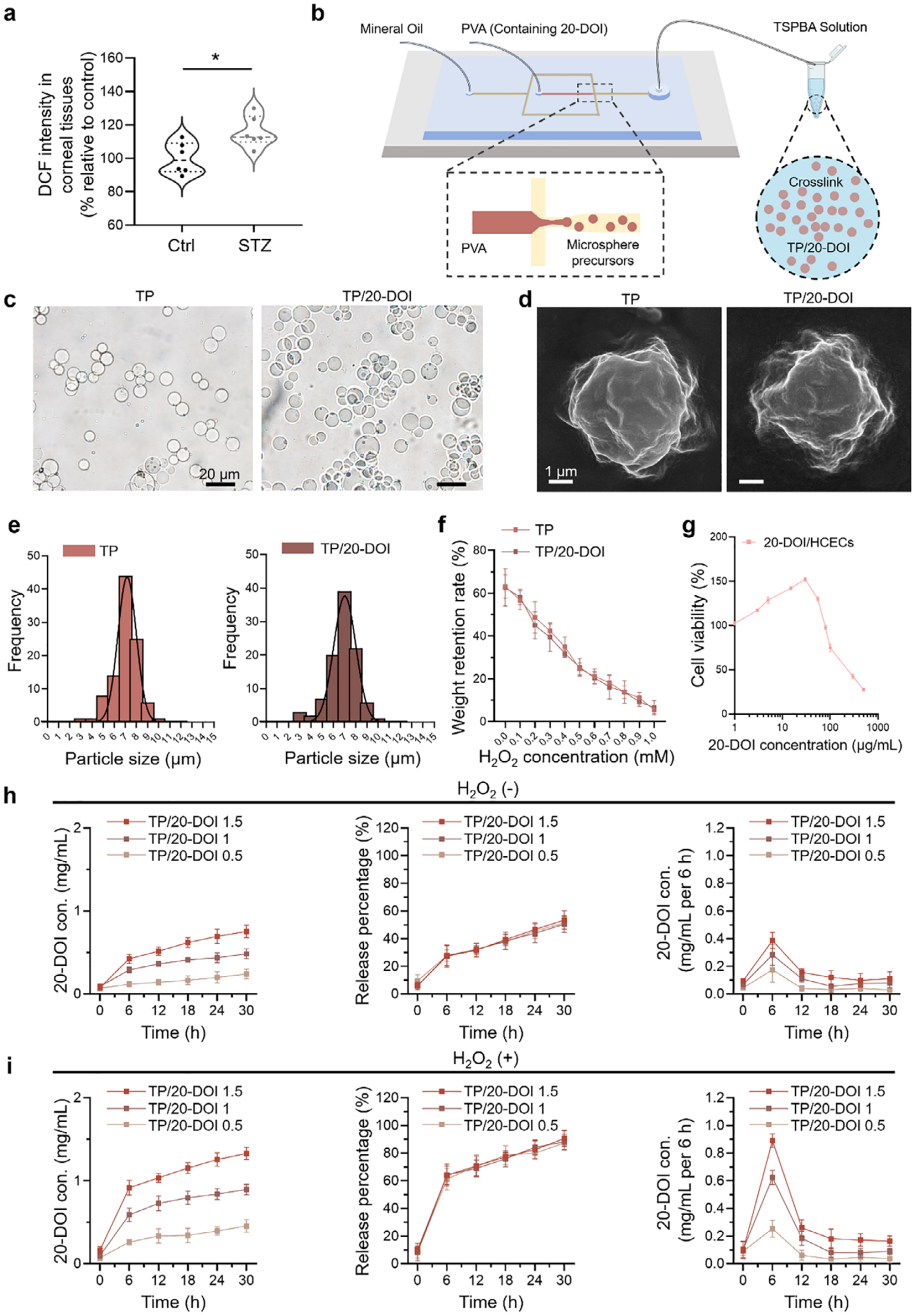
Preparation and controlled release of TP/20‐DOI microspheres. (a) Quantification of intracellular ROS levels in corneal tissue. (b) Fabrication of TP/20‐DOI microspheres via a microfluidic chip. (c) Bright‐field image of TP and TP/20‐DOI microspheres. Scale bars = 20 µm. (d) Scanning electron microscopy images of a single TP and TP/20‐DOI microsphere. Scale bars = 1 µm. (e) Particle size distribution histogram of TP and TP/20‐DOI microspheres. (f) Degradation rate of TP and TP/20‐DOI microspheres. (g) Viability of human corneal epithelial cells (HCECs) after incubation with different concentrations of 20‐DOI. (h) 20‐DOI concentration in simulated body fluid (SBF) solution at 0, 6, 12, 18, 24, and 30 h, measured by mass spectrometry: (Left) Total cumulative concentration of released 20‐DOI; (Middle) Percentage of 20‐DOI released; (Right) Concentration of 20‐DOI released per 6‐h interval. (i) 20‐DOI concentration in H_2_O_2_‐containing SBF solution at 0, 6, 12, 18, 24, and 30 h, measured by mass spectrometry: (Left) Total cumulative concentration of released 20‐DOI; (Middle) Percentage of 20‐DOI released; (Right) Concentration of 20‐DOI released per 6‐h interval. Data are presented as mean ± SD, n = 6. Statistical differences were analyzed by unpaired Student's t‐test. *p < 0.05. TP, TSPBA‐PVA; TP/20‐DOI, TSPBA‐PVA loaded 20‐Deoxyingenol; ROS, reactive oxygen species; SD, standard deviation.

TP/20‐DOI microspheres were fabricated via microfluidic emulsification and chemical cross‐linking (Figure 1b). In this study, TP microspheres refer to the unloaded TSPBA‐PVA carrier, whereas TP/20‐DOI microspheres refer to the 20‐DOI‐loaded formulation. Bright‐field imaging (Figure 1c), scanning electron microscopy (Figure 1d), and particle‐size analysis (Figure 1e) showed that both TP and TP/20‐DOI microspheres were uniformly spherical and exhibited a narrow size distribution, with average diameters of 7.28 ± 0.91 and 6.81 ± 1.21 µm, respectively, and low polydispersity index of 0.016 and 0.032, indicating good size uniformity. Based on the elevated ROS levels observed in diabetic corneal tissue, we next assessed the in vitro degradation behavior of the microsphere system in the presence of H_2_O_2_, which served as a standardized oxidative stimulus to mimic the pathological ROS microenvironment. The degradation rate of TP and TP/20‐DOI microspheres increased in a H_2_O_2_ concentration‐dependent manner (Figure 1f), confirming their ROS‐responsive degradation property. Cytotoxicity assays in human corneal epithelial cells (HCECs) indicated dose‐dependent effects of 20‐DOI, with low toxicity at low concentrations but a sharp increase above 67.64 µg mL^−1^ (the maximum non‐toxic concentration) (Figure 1g). To define the actual amount of drug incorporated into the delivery system before analyzing release behavior, we measured the drug‐loading (DL) content and encapsulation efficiency (EE) of TP/20‐DOI microspheres prepared with 0.5, 1, and 1.5 mg mL^−1^ 20‐DOI. The corresponding DL and EE values are summarized in Table S1, clarifying how the initial feeding concentration translated into the final drug content of the microspheres and providing a basis for the subsequent release‐profile analysis.

Release profiles were further evaluated using microspheres prepared with 0.5, 1, or 1.5 mg mL^−1^ 20‐DOI (denoted as TP/20‐DOI 0.5, TP/20‐DOI 1, and TP/20‐DOI 1.5). In the absence of H_2_O_2_, TP/20‐DOI microspheres exhibited slow release over 30 h, with the released 20‐DOI concentration positively correlating with the loading amount (Figure 1h, left). Cumulative release gradually increased, reaching 40–50% after 30 h (Figure 1h, middle). All groups showed similar release trends, including an initial burst phase. When calculating the average release levels per 6 h, the initial burst phase (i.e., data from the first 6 h) was first excluded. The measured average release levels were as follows: TP/20‐DOI 1.5 at 120.96 ± 45.87 µg mL^−1^; TP/20‐DOI 1 at 52.56 ± 23.27 µg mL^−1^; and TP/20‐DOI 0.5 at 30.45 ± 19.38 µg mL^−1^. Among these formulations, only TP/20‐DOI 1 showed a mean released concentration close to the maximum non‐toxic concentration of 20‐DOI (67.64 µg mL^−1^; Figure 1h, right).

In the presence of H_2_O_2_, 20‐DOI release from TP/20‐DOI microspheres efficiency increased substantially (Figure 1i). The release concentration remained positively correlated with loading (Figure 1i, left), and cumulative release reached about 95% within 30 h (Figure 1i, middle). A pronounced burst release was observed at 6 h, followed by a decline and subsequent stabilization. In simulated body fluid containing H_2_O_2_, the average release levels per 6 h of the drug‐loaded microspheres were: TP/20‐DOI 1.5 at 193.69 ± 63.47 µg mL^−1^; TP/20‐DOI 1 at 47.85 ± 18.41 µg/mL; and TP/20‐DOI 0.5 at 35.32 ± 27.69 µg mL^−1^. Notably, among the three formulations, only TP/20‐DOI 1 yielded a mean released concentration close to the maximum non‐toxic concentration of 20‐DOI (67.64 µg mL^−1^; Figure 1i, right).

These results confirm the successful preparation of ROS‐responsive 20‐DOI‐loaded microspheres. Importantly, the formulation does not merely encapsulate 20‐DOI, but converts the oxidative feature of the diabetic wound microenvironment into a controlled‐release cue. The release profile of TP/20‐DOI 1 microspheres within a non‐toxic range, thereby providing a material basis for controlled local delivery under oxidative stress.

The ocular formulation properties of the final TP/20‐DOI microsphere suspension were further evaluated to assess its suitability for topical ophthalmic administration. The suspension exhibited shear‐thinning behavior, satisfactory physical stability, redispersibility, and dosing uniformity, together with favorable mucoadhesion and prolonged resistance to tear wash‐off, supporting its applicability as an ophthalmic topical formulation (Figure S1 and Table S2).

### 2.2 TP/20‐DOI Microspheres Promote M2 Polarization of Macrophages Under High‐Glucose Conditions

Given the critical role of macrophage polarization in shaping post‐injury inflammation in diabetic corneas [15], we systematically evaluated whether TP/20‐DOI microspheres could modulate macrophage phenotype under high‐glucose stress. Cytotoxicity assays confirmed that the selected concentrations did not impair RAW264.7 cell viability or proliferation (Figure S2), allowing the subsequent polarization analyses to be interpreted independently of overt cytotoxic effects.

Under 35 mM high‐glucose (HG) conditions, used to model a diabetic‐like microenvironment, with interleukin‐10 (IL‐10)‐treated cells serving as a positive control for M2 polarization, 20‐DOI, TP microspheres, and TP/20‐DOI microspheres all increased the expression of the M2‐associated markers CD206 and arginase 1 (ARG1), while decreasing the M1‐associated markers CD86 and inducible nitric oxide synthase (iNOS) (Figure 2a–c). This pattern indicates that these treatments shifted macrophages away from a pro‐inflammatory state toward a reparative M2‐like phenotype. Notably, the induction of CD206 and ARG1 was more pronounced in the TP/20‐DOI and 20‐DOI groups than in the TP‐alone group, suggesting that 20‐DOI is a major contributor to the polarization‐driving effect.

**FIGURE 2:**
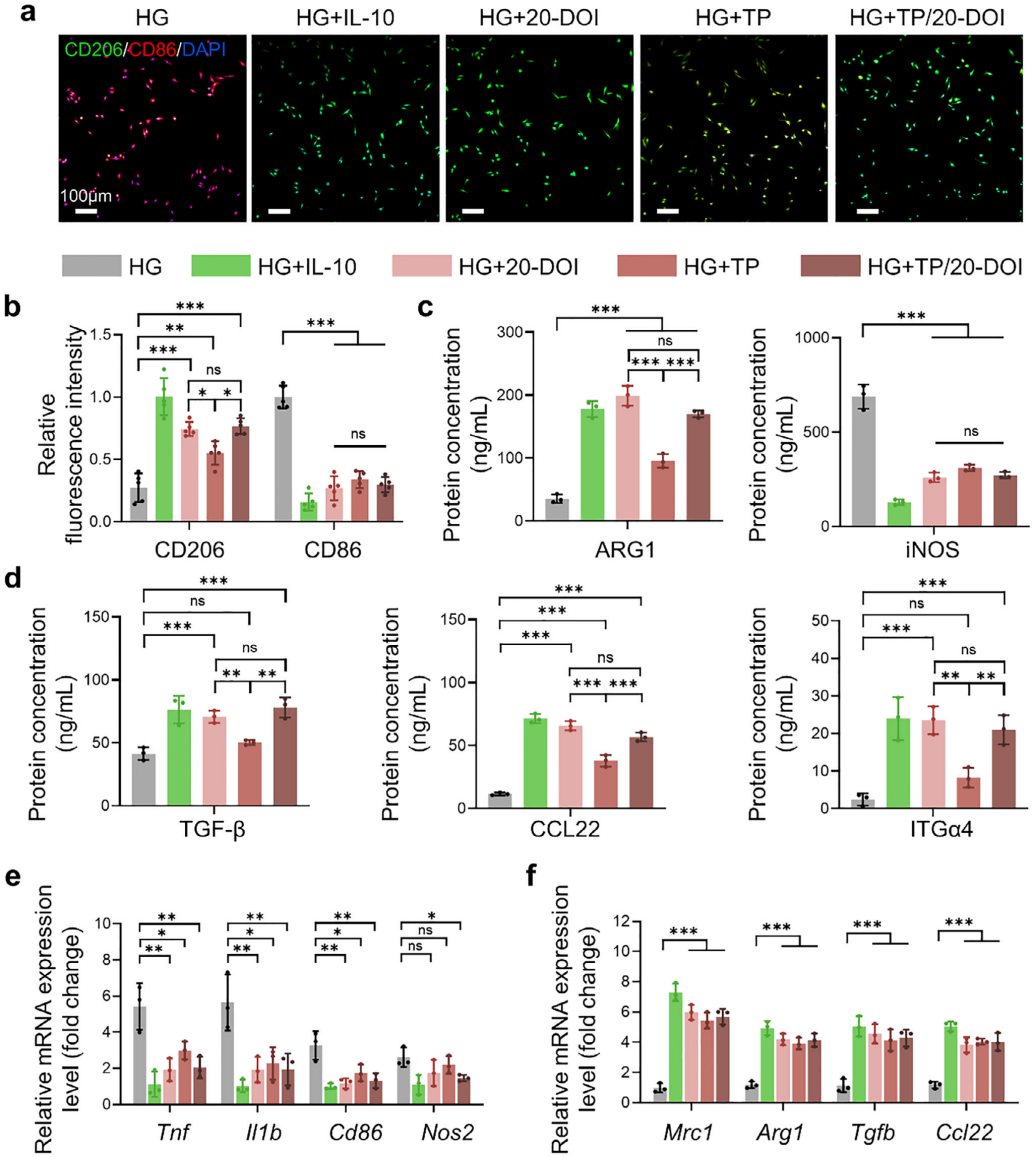
Effects of 20‐DOI, TP microspheres, and TP/20‐DOI microspheres on RAW264.7 cell polarization. (a,b) Immunofluorescence staining of macrophage polarization markers (green: CD206, red: CD86) and quantitative analysis (n = 5). (c,d) ELISA analysis of ARG1, iNOS, TGF‐β, CCL22, and ITGα4 (n = 3). (e,f) RT‐qPCR analysis of *Tnf*, *Il1b*, *Cd86*, *Nos2*, *Mrc1*, *Arg1*, *Tgfb* and *Ccl22* expression (n = 3). Data are presented as mean ± SD. Statistical differences were analyzed by one‐way ANOVA followed by Tukey's multiple comparisons test. ns: *p* > 0.05, *: *p* < 0.05, **: *p* < 0.01, ***: *p* < 0.001. 20‐DOI, 20‐Deoxyingenol; TP, TSPBA‐PVA; TP/20‐DOI, TSPBA‐PVA loaded 20‐Deoxyingenol; ARG1, arginase 1; iNOS, inducible nitric oxide synthase; TGF‐β, transforming growth factor beta; ITGα4, integrin alpha 4; RT‐qPCR, reverse transcription quantitative polymerase chain reaction; *Tnf*, tumor necrosis factor; *Il1b*, interleukin 1beta; SD, standard deviation; ns, no significance.

We next assessed functional molecules associated with this phenotypic transition. TP/20‐DOI microspheres and 20‐DOI significantly elevated transforming growth factor beta (TGF‐β), CCL22, and integrin alpha 4 (ITGα4) levels (Figure 2d). In the context of corneal repair, the increase in TGF‐β is biologically relevant because TGF‐β signaling is closely involved in wound‐healing responses [40]. The elevation of CCL22 is consistent with an M2‐like immunoregulatory phenotype, as CCL22 is a recognized M2‐associated macrophage chemokine and can contribute to a pro‐resolution microenvironment through CCR4‐dependent regulatory circuits [41, 42]. Increased ITGα4 further suggests altered adhesion‐ and tissue‐interaction properties [43] that may favor constructive communication between macrophages and the injured microenvironment. Therefore, these changes are best understood not as isolated marker fluctuations, but as a coordinated molecular pattern more compatible with inflammatory resolution and tissue repair.

Consistent with the protein‐level findings, reverse transcription quantitative polymerase chain reaction (RT‐qPCR) further showed that 20‐DOI, TP microspheres, and TP/20‐DOI microspheres significantly reduced the expression of the M1 marker *Cd86* and the pro‐inflammatory genes, tumor necrosis factor (*Tnf*) and *Il1b* (Figure 2e), while increasing the expression of the M2 marker *Mrc1* and the anti‐inflammatory or repair‐associated genes *Arg1*, *Tgfb*, and *Ccl22* (Figure 2f). Thus, TP/20‐DOI microspheres simultaneously suppress the inflammatory arm of macrophage activation and reinforces the reparative arm under diabetic stress conditions.

Together, these findings demonstrate that TP/20‐DOI microspheres effectively promote a reparative macrophage program, rather than merely altering individual polarization markers, thereby creating an immune microenvironment more favorable for inflammatory resolution and diabetic corneal repair.

### 2.3 TP/20‐DOI Microspheres Activate Autophagy and Oxidative Phosphorylation in Macrophages Under High‐Glucose Conditions

To investigate the molecular basis by which TP/20‐DOI microspheres regulate macrophage phenotype, we performed mRNA sequencing in RAW264.7 cells. Both 20‐DOI and TP microspheres altered the global transcriptional landscape under high‐glucose conditions, as shown by the differential expression heatmap (Figure 3a), indicating that both the bioactive compound and the carrier contribute biologically active signals rather than acting as passive components.

**FIGURE 3:**
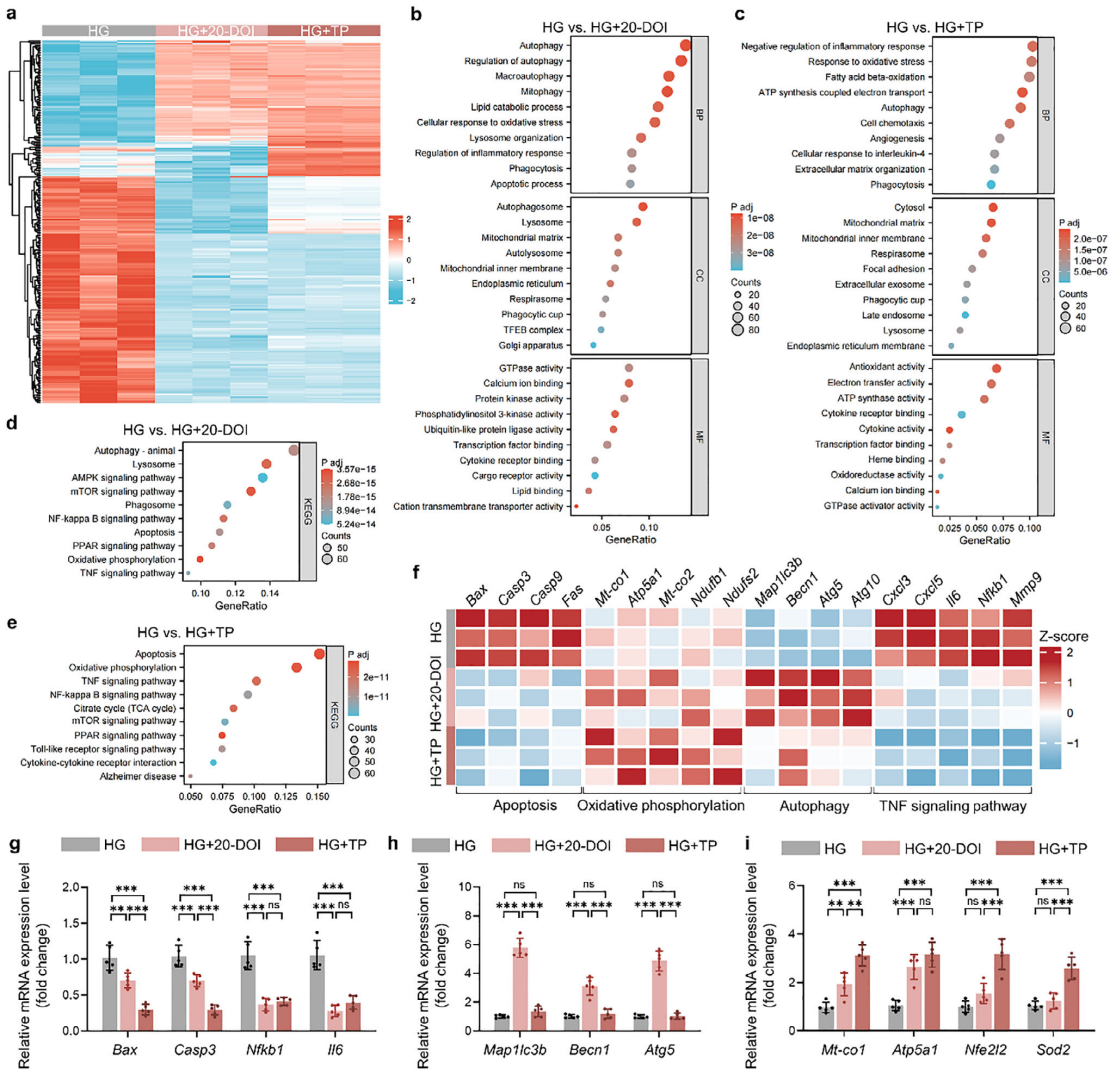
mRNA sequencing after 20‐DOI and TP microsphere treatments. (a) Heatmap of DEGs among the HG, HG+20‐DOI and HG+TP groups. (b) GO enrichment analysis between the HG+20‐DOI and HG groups. (c) GO enrichment analysis between the HG+TP and HG groups. (d) KEGG pathway enrichment analysis between the HG+20‐DOI and HG groups. (e) KEGG pathway enrichment analysis between the HG+TP and HG groups. (f) Heatmap of representative genes related to apoptosis, oxidative phosphorylation, autophagy, and TNF signaling pathway. (g) RT‐qPCR of apoptosis‐ and inflammation‐related genes (n = 5). (h) RT‐qPCR of autophagy‐related genes (n = 5). (i) RT‐qPCR of mitochondrial function‐related genes (n = 5). Data are presented as mean ± SD. Statistical differences were analyzed by one‑way ANOVA followed by Tukey's multiple comparisons test. ns: *p* > 0.05, *: *p* < 0.05, **: *p* < 0.01, ***: *p* < 0.001. 20‐DOI, 20‐Deoxyingenol; TP, TSPBA‐PVA; DEGs, differential expression genes; HG, high glucose; GO, Gene Ontology; KEGG, Kyoto Encyclopedia of Genes and Genomes; TNF, tumor necrosis factor; RT‐qPCR, reverse transcription quantitative polymerase chain reaction; SD, standard deviation; ns, no significance.

Gene Ontology (GO) enrichment analysis revealed that genes regulated by 20‐DOI were predominantly associated with autophagy‐related processes (Figure 3b), whereas genes regulated by TP microspheres were enriched in pathways related to inflammatory regulation, oxidative stress response, and ATP synthesis‐coupled electron transport (Figure 3c). This distinction suggests that 20‐DOI and TP microspheres may play complementary roles: 20‐DOI more strongly engages intracellular quality‐control pathways, whereas TP microspheres more prominently influence inflammation and redox.

Kyoto Encyclopedia of Genes and Genomes (KEGG) pathway analysis further supported this interpretation. In the 20‐DOI group, enriched pathways included autophagy, lysosome, apoptosis, as well as 5'‐AMP‐activated protein kinase (AMPK), mechanistic target of rapamycin (mTOR), and nuclear factor‐kappa B (NF‐κB) signaling pathways (Figure 3d), indicating that 20‐DOI may counter high‐glucose stress by restoring autophagy while restraining inflammatory and injury‐related signaling. In contrast, treatment of TP microspheres primarily enriched apoptosis, oxidative phosphorylation, the tricarboxylic acid (TCA) cycle, and inflammatory signaling such as tumor necrosis factor (TNF) and NF‐κB (Figure 3e), suggesting that the carrier itself contributes to metabolic stabilization and inflammatory control.

Consistent with the transcriptomic analysis, the heatmap of representative genes showed that both 20‐DOI and TP microspheres downregulated apoptosis‐ and TNF signaling‐related genes while upregulating oxidative phosphorylation‐ and autophagy‐related genes (Figure 3f). Importantly, the autophagy‐related increase was more pronounced in the 20‐DOI group, indicating that autophagy restoration is a particularly prominent component of 20‐DOI activity.

RT‐qPCR further validated these patterns. Both 20‐DOI and TP microspheres reduced the expression of pro‐apoptotic markers, BCL2‐associated X protein (*Bax*) and Caspase‐3 (*Casp3*) and inflammatory mediators (*Nfkb1*, *Il6*) (Figure 3g), indicating alleviation of high‐glucose‐induced cellular stress. However, their mechanistic contributions were not identical. 20‐DOI more strongly increased the expression of autophagy‐related markers, microtubule‐associated protein 1 light chain 3 beta (*Map1lc3b*), beclin‐1 (*Becn1*), and autophagy protein 5 (*Atg5*) (Figure 3h), whereas TP microspheres significantly promoted the expression of mitochondrial and antioxidant‐related markers, cytochrome c oxidase subunit 1 (*Mtco1*), ATP synthase F1 subunit alpha (*Atp5a1*), nuclear factor erythroid 2‐related factor 2 (*Nfe2l2*), and superoxide dismutase 2 (*Sod2*) (Figure 3i).

Taken together, these data suggest that TP microspheres and 20‐DOI counteract high‐glucose‐induced macrophage dysfunction through complementary mechanisms: TP microspheres mainly support redox balance, whereas 20‐DOI more strongly restores autophagic activity. Their combination therefore provides a mechanistic basis for simultaneous recovery of cellular homeostasis, metabolic competence, and inflammatory restraint.

### 2.4 TP/20‐DOI Microspheres Reprogram Macrophage Metabolism Toward Oxidative Phosphorylation to Support M2 Polarization Under High‐Glucose Conditions

Because autophagy is tightly linked to mitochondrial quality control, we next examined whether TP/20‐DOI microspheres also reprogram macrophage metabolism under high‐glucose stress. Oxygen consumption rate (OCR) analysis showed that, compared with the high‐glucose group, 20‐DOI, TP microspheres, and TP/20‐DOI microspheres significantly increased basal respiration, ATP production, and maximal respiratory capacity, while reducing proton leak (Figure 4a‐e). These changes indicate improved mitochondrial efficiency rather than a simple increase in energy consumption.

**FIGURE 4:**
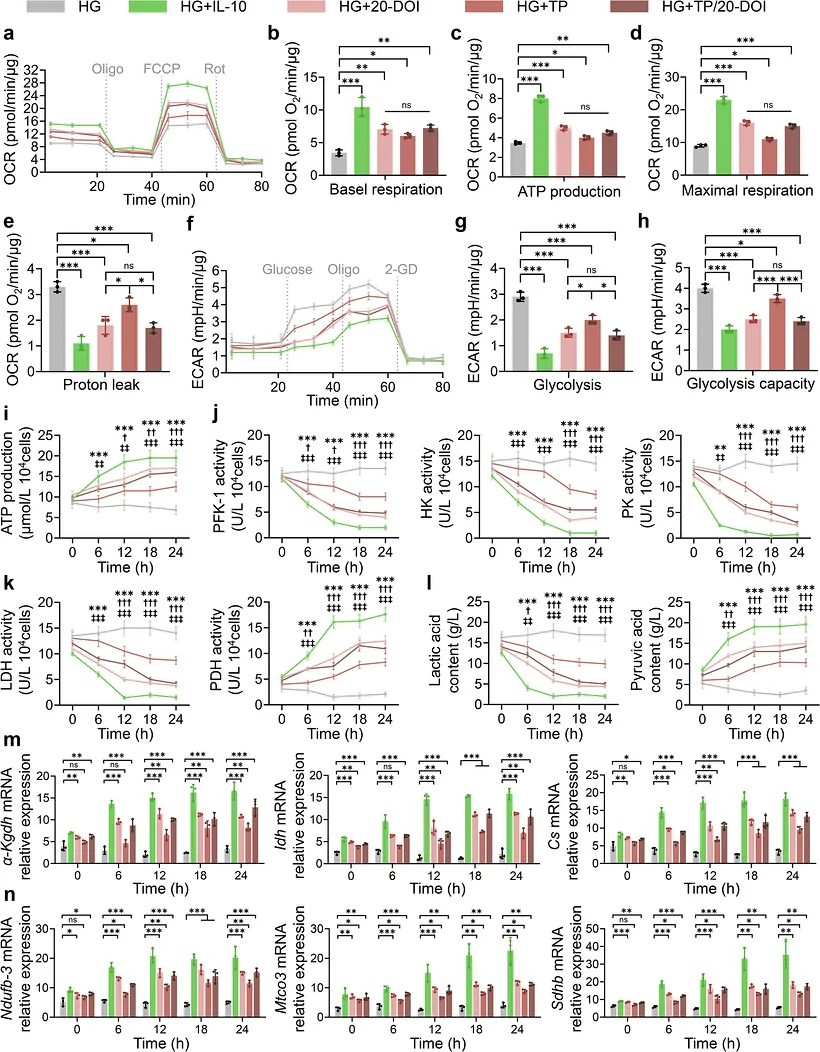
Effects of 20‐DOI, TP microspheres, and TP/20‐DOI microspheres on macrophage metabolism. a–e) OCR parameters measured by Seahorse metabolic analysis (n = 3). (f–h) ECAR analysis obtained via Seahorse metabolic analysis (n = 3). (i) ATP production measured at different time points post‑treatment (n = 3). (j) Activities of PFK‐1, HK and PK measured at different time points post‐treatment (n = 3). (k) Activities of LDH and PDH (n = 3). (l) Content of lactate and pyruvate (n = 3). (m) RT‐qPCR analysis of *α‐Kgdh*, *Idh* and *Cs* expression (n = 3). (n) RT‐qPCR analysis of *Ndufb3*, *Mtco3* and *Sdhb* expression (n = 3). Data are presented as mean ± SD. Statistical differences were analyzed by one‐way ANOVA followed by Tukey's multiple comparisons test. ns: *p* > 0.05; *,†,‡: *p* < 0.05; **,††,‡‡: *p* < 0.01; ***,†††,‡‡‡: *p* < 0.001; (**i**‑**l**) *: HG+20‐DOI group vs. HG group, †: HG+TP group vs. HG group; ‡: HG+TP/20‐DOI group vs. HG group. 20‐DOI, 20‐Deoxyingenol; TP, TSPBA‐PVA; TP/20‐DOI, TSPBA‐PVA loaded 20‐Deoxyingenol; OCR, Oxygen Consumption Rate; ECAR, Extracellular Acidification Rate; PFK‐1, phosphofructokinase‐1; HK, hexokinase; PK, pyruvate kinase; LDH, lactate dehydrogenase; PDH, pyruvate dehydrogenase; RT‐qPCR, reverse transcription quantitative polymerase chain reaction; *α‐Kgdh*, alpha‐ketoglutarate dehydrogenase; *Idh*, isocitrate dehydrogenase; *Cs*, citrate synthase; *Ndufb3*, NADH dehydrogenase [ubiquinone] 1 beta subcomplex subunit 3; *Mtco3*, cytochrome c oxidase subunit 3; *Sdhb*, succinate dehydrogenase subunit B; SD, standard deviation; ns, no significance.

In parallel, extracellular acidification rate (ECAR) analysis demonstrated that 20‐DOI, TP microspheres, and TP/20‐DOI microspheres reduced both basal glycolysis and glycolytic capacity (Figure 4f‐h). Thus, the metabolic effect of these treatments is not merely to boost ATP generation, but to redirect macrophages away from a glycolysis‐dominant stress state toward a more mitochondria‐dependent bioenergetic state. Importantly, TP/20‐DOI microspheres behaved more similarly to 20‐DOI than to TP microspheres alone in OCR/ECAR readouts, consistent with the strong polarization‐promoting effect observed in Figure 2.

We then examined additional indices of glucose metabolism. Compared with the high glucose group, 20‐DOI, TP microspheres, and TP/20‐DOI microspheres increased ATP production (Figure 4i), reduced the activity of glycolytic enzymes phosphofructokinase 1 (PFK‐1), hexokinase (HK), pyruvate kinase (PK), and lactate dehydrogenase (LDH) (Figure 4j,k), increased pyruvate dehydrogenase (PDH) activity (Figure 4k), decreased lactate accumulation, and increased pyruvic acid content (Figure 4l). These coordinated changes indicate reduced reliance on inflammatory glycolysis and improved entry of carbon substrates into mitochondrial oxidation.

Consistently, the mRNA expression of key TCA cycle enzymes, alpha‐ketoglutarate dehydrogenase (*α‐Kgdh*), isocitrate dehydrogenase (*Idh*), and citrate synthase (*Cs*) and oxidative phosphorylation complex subunits, NADH dehydrogenase [ubiquinone] 1 beta subcomplex subunit 3 (*Ndufb3*), cytochrome c oxidase subunit 3 (*Mtco3*), and succinate dehydrogenase subunit B (*Sdhb*) was upregulated after 20‐DOI, TP microspheres, or TP/20‐DOI microspheres treatment (Figure 4m,n), further supporting restoration of mitochondrial metabolic capacity.

Together, these findings indicate that TP/20‐DOI microspheres re‐establish a metabolic configuration characterized by reduced glycolytic stress and enhanced oxidative phosphorylation. Additional rotenone‐intervention experiments further showed that pharmacological disruption of oxidative phosphorylation attenuated the TP/20‐DOI‐induced M2‐like polarization program and restored a more pro‐inflammatory expression profile, including increased IL1β expression and enhanced expression of the neutrophil‐recruiting chemokines CXCL3 and CXCL5 (Figure S3), thereby providing direct functional support for a causal link between metabolic reprogramming and macrophage polarization.

### 2.5 TP/20‐DOI Microspheres Promote Corneal Wound Healing in Diabetic Mice After Epithelial Abrasion

To evaluate the therapeutic potential of TP/20‐DOI microspheres, we administered 20‐DOI, TP microsphere, or TP/20‐DOI microsphere eye drops to STZ‐induced diabetic mice after corneal epithelial abrasion (Figure 5a). The complete timeline of the animal experiments, including model establishment, treatment administration, and subsequent sample collection and processing, is presented in Figure S4. Fluorescein staining showed that both 20‐DOI and TP/20‐DOI microspheres accelerated wound closure at 24 h post‐injury, with a more pronounced effect in the TP/20‐DOI group (Figure 5b,c). This result indicates that the combined formulation improves the speed of epithelial resurfacing under diabetic conditions. To assess whether the enhanced in vivo efficacy of TP/20‐DOI microspheres was related to prolonged ocular‐surface retention, we tracked Cy5‐labeled 20‐DOI after administration as free eye drops or TP/20‐DOI microsphere eye drops. TP/20‐DOI microspheres persisted longer on the corneal surface than free 20‐DOI, indicating improved ocular‐surface retention (Figure S5), which likely contributes to their superior therapeutic effect in vivo.

**FIGURE 5:**
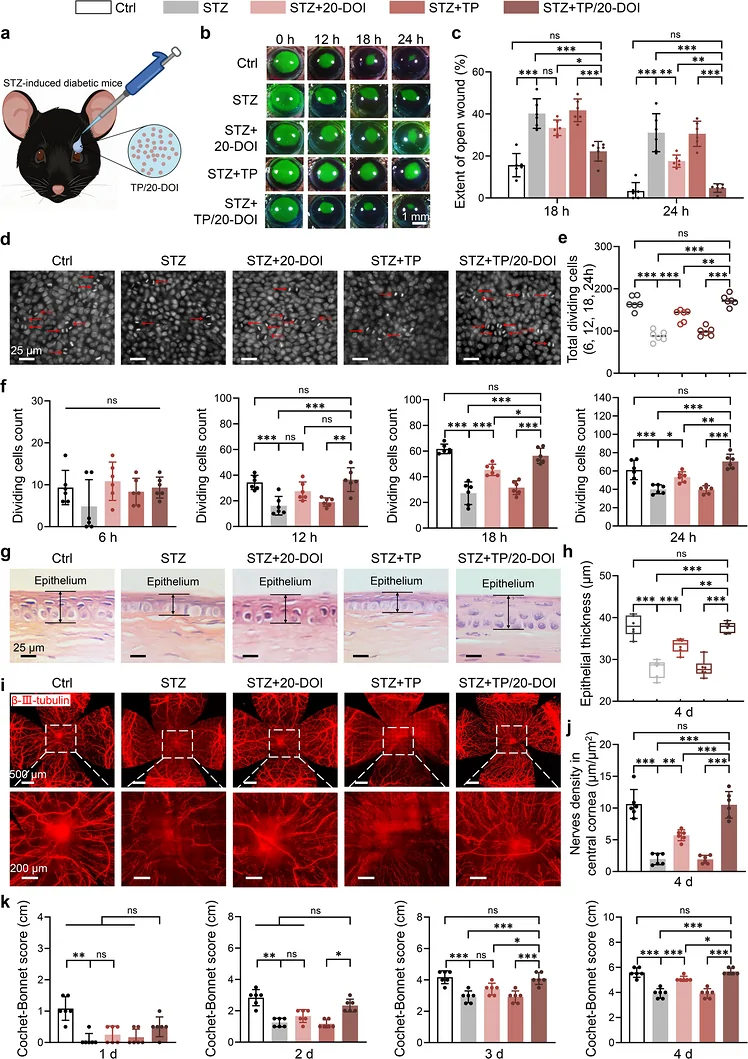
Effects of 20‐DOI, TP microspheres, and TP/20‐DOI microspheres on corneal re‐epithelialization and nerve regeneration in diabetic mice. (a) Schematic illustration of TP/20‐DOI microsphere eyedrops administration to STZ‐induced diabetic mice. (b) Sodium fluorescein staining of corneal wound area. (c) Comparison of the extent of corneal open wound among the five group mice. (d) DAPI staining of corneal epithelial basal cells observed under a 40× objective lens. (e) Comparison of the cumulative number of dividing cells across all time points (6, 12, 18, 24 h) after epithelial abrasion among the five group mice. (f) Time‐course analysis of dividing epithelial basal cells in the five group mice from 6 to 24 h after abrasion. (g) H&E staining of paraffin‐embedded corneal sections at 4 days after epithelial abrasion. (h) Comparison of epithelial thickness among the five group mice. (i) Immunostaining of corneal nerve fibers at 4 days after epithelial abrasion with anti‐β‐III tubulin antibody. (j) Comparison of nerve density in central cornea (1 mm × 1 mm) among the five group mice. (k) Difference in corneal sensitivity in the five group mice at 1, 2, 3, and 4 days following corneal abrasion. Data are presented as mean ± SD. Statistical differences were analyzed by one‐way ANOVA followed by Tukey's multiple comparisons test, *n* = 6 mice per group. ns: *p* > 0.05, *: *p* < 0.05, **: *p* < 0.01, ***: *p* < 0.001. 20‐DOI, 20‐Deoxyingenol; TP, TSPBA‐PVA; TP/20‐DOI, TSPBA‐PVA loaded 20‐Deoxyingenol; STZ, streptozotocin; DAPI, 4',6‐diamidino‐2‐phenylindole; H&E, hematoxylin and eosin; SD, standard deviation; ns, no significance.

Whole‐mount corneal staining further demonstrated markedly increased epithelial basal‐cell proliferation in TP/20‐DOI‐treated mice relative to the other groups (Figure 5d–f). Histological analysis (H&E staining) showed that TP/20‐DOI microspheres also significantly increased corneal epithelial thickness at day 4 after injury compared with 20‐DOI or TP alone (Figure 5g,h). To further assess epithelial barrier‐related structural recovery, we performed zonula occludens‐1 (ZO‐1) staining at 24 h after corneal injury. Compared with the STZ group, both 20‐DOI and TP/20‐DOI increased the ZO‐1‐positive junctional length in the corneal epithelium, with a more pronounced effect observed in the TP/20‐DOI group (Figure S6a,b). Therefore, TP/20‐DOI microspheres improve not only the rate of epithelial defect closure, but also epithelial reconstruction and barrier‐related junctional integrity. At later time points, quantitative measurement of corneal light transmittance at day 14 showed no significant differences among the treatment groups, indicating comparable recovery of corneal transparency (Figure S7a). In addition, α‐SMA immunofluorescence staining at days 7 and 14 showed no positive staining in any group (Figure S7b,c), suggesting that overt fibrosis was not induced in this model under our experimental conditions. This observation is consistent with previous reports that corneal epithelial debridement alone is insufficient to trigger fibrosis [44].

Because diabetic keratopathy is also characterized by impaired corneal innervation, we further assessed nerve repair. TP/20‐DOI microspheres increased corneal nerve density more strongly than either 20‐DOI or TP microspheres alone (Figure 5i,j), and this structural recovery was accompanied by improved corneal sensitivity (Figure 5k). These findings indicate that the therapeutic effect of TP/20‐DOI microspheres extends beyond epithelial closure to functional neural recovery, which is clinically important because restoration of corneal sensation is closely linked to long‐term ocular‐surface integrity. We further included basic fibroblast growth factor (bFGF) as a clinically relevant reference treatment, given its documented pro‐reparative effects on the corneal epithelium and its reported benefit in corneal sensory recovery [45, 46, 47]. TP/20‐DOI microspheres showed greater efficacy than bFGF in accelerating corneal epithelial wound closure; however, no significant difference was observed between the two groups in terms of nerve repair (Figure S8).

To determine whether TP/20‐DOI microspheres directly promote corneal epithelial wound closure in diabetic mice. HCEC scratch assay was performed and showed no significant differences in wound closure among HG, HG + 20‐DOI, HG + TP, and HG + TP/20‐DOI groups at 12 or 24 h (Figure S9), suggesting that the in vivo therapeutic benefit was unlikely to arise primarily from a direct epithelial pro‐migratory effect. Therefore, we next investigated whether the therapeutic benefit of TP/20‐DOI microspheres were associated with inflammatory reprogramming.

### 2.6 TP/20‐DOI Microspheres Activates Autophagy and Decreases Inflammation in Diabetic Corneas

Impaired autophagy and excessive inflammation are major contributors to delayed diabetic corneal healing [13, 14, 15]. Based on the in vitro transcriptomic results, we next asked whether TP/20‐DOI microspheres could restore these processes in vivo. In diabetic corneas, TP/20‐DOI microspheres increased the expression of the autophagy‐related genes microtubule‐associated protein 1 light chain 3 beta (*Map1lc3b*), *Atg10*, and *Atg14* (Figure 6a), and monodansylcadaverine (MDC) staining further confirmed enhanced autophagic activity in corneal cells after treatment (Figure 6b). These results indicate that TP/20‐DOI microspheres reactivate an autophagy‐associated homeostatic program that is otherwise suppressed in diabetic tissue.

**FIGURE 6:**
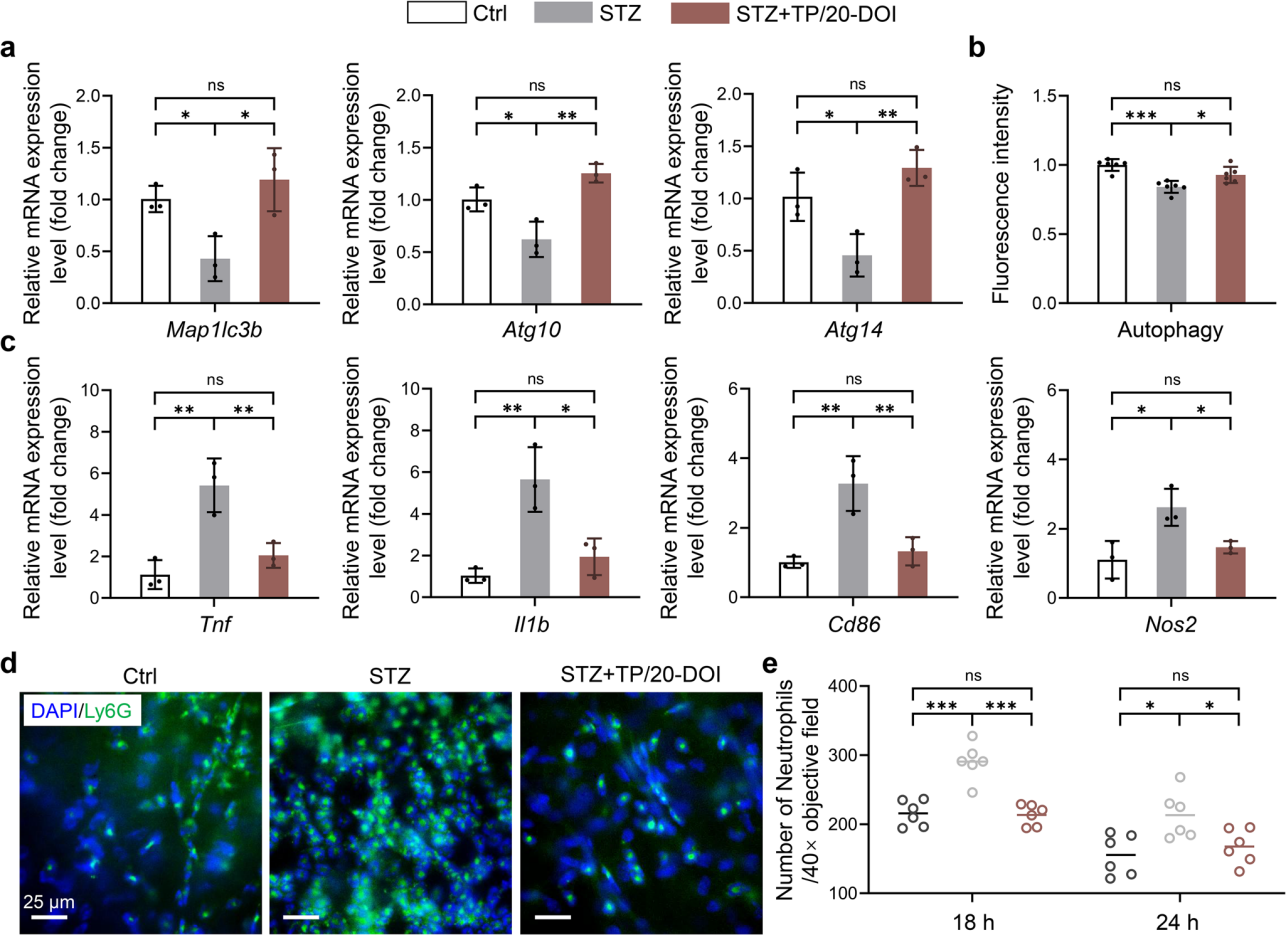
Evaluation of the effects of TP/20‐DOI microspheres on corneal autophagy and inflammation after epithelial abrasion in diabetic mice. (a) RT‐qPCR analysis of *Map1lc3b*, *Atg10*, and *Atg14* gene expression in corneas of Control, STZ, STZ administered TP/20‐DOI mice. (b) Detection of corneal cell autophagy in the three group of mice with MDC. (c) RT‐qPCR analysis of *Tnf*, *Il1b*, *Cd86*, and *Nos2* gene expression in corneas of the three group of mice. (d) Immunostaining of corneas with DAPI and anti‐Ly6G antibody conjugated with FITC observed under 40× objective lens. (e) Analysis of infiltrated neutrophils to corneas in the three group of mice at 18 and 24 h after abrasion. Data are presented as mean ± SD. Statistical differences were analyzed by one‐way ANOVA followed by Tukey's multiple comparisons test; *n* = 3 independent experiments (2 mice per experiment in each group) (a,c); *n* = 6 mice per group (b,e). ns: *p* > 0.05, *: *p* < 0.05, **: *p* < 0.01, ***: *p* < 0.001. TP/20‐DOI, TSPBA‐PVA loaded 20‐Deoxyingenol; RT‐qPCR, reverse transcription quantitative polymerase chain reaction; *Map1lc3b*, microtubule‐associated protein 1 light chain 3 beta; *Atg10*, autophagy protein 10; STZ, streptozotocin; MDC, monodansylcadaverine; *Tnf*, tumor necrosis factor; *Il1b*, interleukin 1beta; *Nos2*, inducible nitric oxide synthase; DAPI, 4',6‐diamidino‐2‐phenylindole; FITC, fluorescein isothiocyanate; SD, standard deviation; ns, no significance.

We then assessed inflammatory status in the same tissue context. Expression of the M1‐associated inflammatory genes *Tnf*, *Il1b*, *Cd86*, and *Nos2* was elevated in diabetic corneas but significantly reduced by TP/20‐DOI microsphere treatment (Figure 6c). In parallel, neutrophil accumulation, which was markedly increased at 18 and 24 h after injury in diabetic corneas, was substantially attenuated by TP/20‐DOI microspheres (Figure 6d,e). Thus, restoration of autophagy in the diabetic cornea is accompanied by a clear dampening of the persistent inflammatory tone that otherwise compromises healing.

Together, these findings suggest that TP/20‐DOI microspheres do not merely alter isolated molecular readouts in vivo, but rebalance the diabetic wound environment toward a state characterized by greater cellular homeostasis and lower inflammatory burden.

### 2.7 TP/20‐DOI Microspheres Attenuate Neutrophil Infiltration in Diabetic Corneal Wounds via Autophagy‐Dependent Suppression of Macrophage‐Derived CXCL3/5

Given the close interplay between autophagy and inflammatory output [48, 49], we next tested whether the anti‐inflammatory effect of TP/20‐DOI microspheres in diabetic corneas depends on autophagy activation. When diabetic mice receiving TP/20‐DOI microspheres were co‐treated with the autophagy inhibitor Spautin‐1, the expression of M1‐associated inflammatory genes (*Tnf*, *Il1b*, *Cd86*, *Nos2*) increased again (Figure 7a), and neutrophil infiltration into the injured cornea was also restored (Figure 7b,c). These data indicate that the anti‐inflammatory action of TP/20‐DOI microspheres is not independent of autophagy, but functionally depends on it.

**FIGURE 7:**
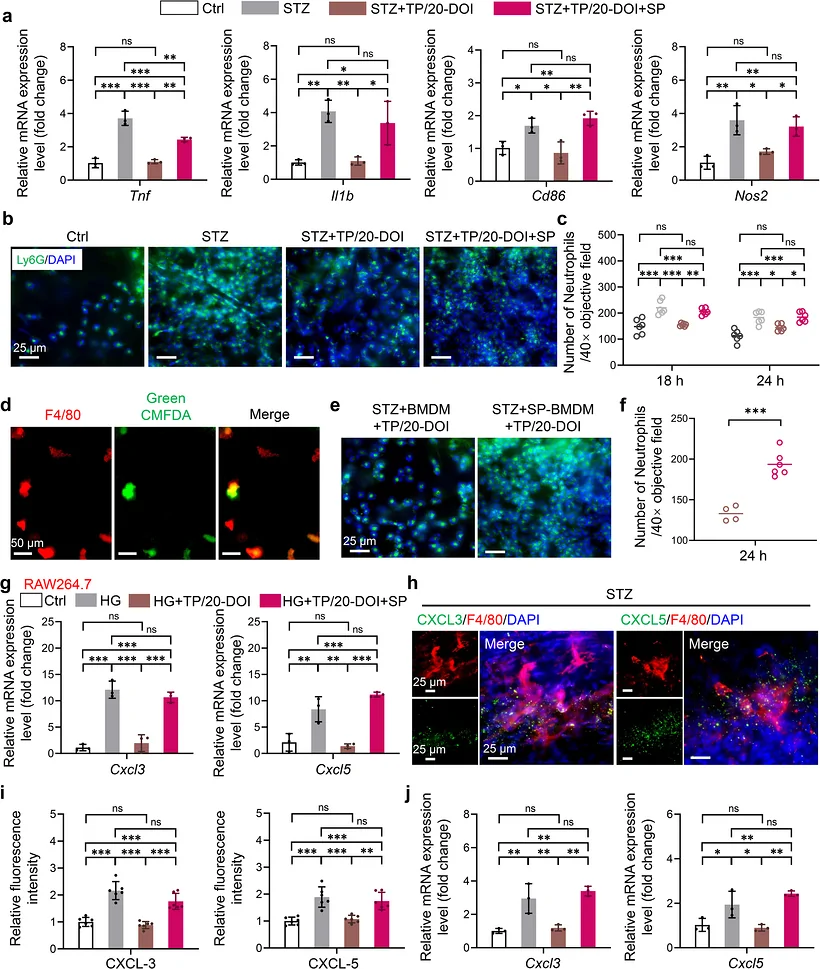
Effects of Spautin‐1 on corneal inflammation after epithelial abrasion in diabetic mice. (a) RT‐qPCR analysis of *Tnf*, *Il1b*, *Cd86*, and *Nos2* gene expression in corneas of control, STZ, STZ + TP/20‐DOI, STZ + TP/20‐DOI+SP mice. (b) Immunostaining of corneas with DAPI and anti‐Ly6G antibody observed under 40× objective lens. (c) Analysis of infiltrated neutrophils to corneas in each group of mice at 18 and 24 h after abrasion. (d) Immunostaining of corneas with anti‐F4/80 antibody. BMDMs were labeled with CellTracker Green CMFDA Dye. (e) Immunostaining of corneas with DAPI and anti‐Ly6G antibody observed under 40× objective lens. (f) Analysis of infiltrated neutrophils to corneas in STZ mice receiving vehicle‐treated BMDMs plus TP/20‐DOI eye drops and STZ mice receiving SP‐treated BMDMs plus TP/20‐DOI eye drops at 24 h after epithelial abrasion. (g) RT‐qPCR analysis of *Cxcl3* and *Cxcl5* gene expression in each group of RAW264.7 cells. (h) Immunostaining of diabetic corneas with DAPI, anti‐CXCL3/CXCL5 and anti‐F4/80 antibody under 40× objective lens. (i) Detection of relative fluorescence intensity of CXCL3/CXCL5 in corneas from each group of mice. (j) RT‐qPCR analysis of *Cxcl3* and *Cxcl5* gene expression in corneas of each group mice. Data are presented as mean ± SD. Statistical differences were analyzed by one‐way ANOVA followed by Tukey's multiple comparisons test or unpaired Student's t‐test; *n* = 3 independent experiments (2 mice per experiment in each group) (a,j); *n* = 4‐6 mice per group (c,f,i). ns: *p* > 0.05, *: *p* < 0.05, **: *p* < 0.01, ***: *p* < 0.001. RT‐qPCR, reverse transcription quantitative polymerase chain reaction; *Tnf*, tumor necrosis factor; *Il1b*, interleukin 1beta; *Nos2*, inducible nitric oxide synthase; STZ, streptozotocin; TP/20‐DOI, TSPBA‐PVA loaded 20‐Deoxyingenol; SP: spautin‐1; DAPI, 4',6‐diamidino‐2‐phenylindole; BMDMs, bone marrow‐derived macrophages; *Cxcl3*, C‐X‐C motif chemokine ligand 3; SD, standard deviation; ns, no significance.

To further determine whether macrophage autophagy contributes to the therapeutic effect of TP/20‐DOI microspheres in vivo, we performed adoptive transfer experiments using macrophages with inhibited autophagy. F4/80^+^ bone marrow‐derived macrophages (BMDMs) isolated from diabetic mice were treated ex vivo with Spautin‐1 or vehicle, followed by adoptive transfer into diabetic recipient mice via tail vein injection at 0 and 12 h after corneal epithelial injury (1 × 10^6^ cells per mouse per injection). Fluorescence tracing with CellTracker Green CMFDA Dye confirmed that the transferred BMDMs successfully migrated to the injured cornea (Figure 7d). Notably, transfer of Spautin‐1‐treated BMDMs significantly weakened the inhibitory effect of TP/20‐DOI microspheres on corneal neutrophil infiltration (Figure 7e,f). These findings provide direct functional evidence that macrophage autophagy is required for the anti‐inflammatory action of TP/20‐DOI microspheres in diabetic corneal injury.

Previous corneal studies have shown that inflammatory macrophages help drive neutrophil accumulation after epithelial injury [15, 16, 17]. We next investigated the mechanism by which autophagy‐dependent macrophage function regulates neutrophil recruitment. In our transcriptomic dataset, 20‐DOI and TP microspheres reduced the expression of the neutrophil‐associated chemokines *Cxcl3* and *Cxcl5* under high‐glucose conditions (Figure 3f). Notably, *Nfkb1* expression was also decreased in parallel, raising the possibility that NF‐κB signaling contributes to the suppression of these chemokines. RT‐qPCR further confirmed that TP/20‐DOI microspheres decreased *Cxcl3* and *Cxcl5* expression in high‐glucose‐cultured RAW264.7 cells, whereas this effect was reversed by Spautin‐1 (Figure 7g). To further test whether NF‐κB signaling is involved in this process, we performed a functional rescue experiment using TNF‐α to reactivate NF‐κB signaling. TP/20‐DOI microspheres significantly reduced the p‐p65/total p65 ratio, confirming inhibition of NF‐κB activation. Addition of TNF‐α restored the p‐p65/total p65 ratio and reversed the TP/20‐DOI‐induced reductions in CXCL3 and CXCL5 secretion (Figure S10). These results support the idea that inhibition of NF‐κB signaling contributes, at least in part, to the suppression of CXCL3 and CXCL5 by TP/20‐DOI microspheres.

In vivo, immunofluorescence staining showed that corneal macrophages expressed CXCL3 and CXCL5 (Figure 7h). Both the fluorescence intensity and mRNA expression of CXCL3 and CXCL5 were reduced in TP/20‐DOI‐treated diabetic corneas but increased again after autophagy inhibition (Figure 7i,j). Therefore, TP/20‐DOI microspheres appear to restrain neutrophil recruitment not merely by broadly suppressing inflammation, but by limiting the chemokine output of macrophages through an autophagy‐dependent mechanism.

Collectively, these findings provide a mechanistic bridge between intracellular macrophage reprogramming and extracellular inflammatory resolution: TP/20‐DOI‐induced autophagy suppresses macrophage‐derived CXCL3/5, thereby reducing secondary neutrophil infiltration into diabetic corneal wounds.

### 2.8 Autophagy Activation Mediates TP/20‐DOI‐Induced Corneal Wound Healing in Diabetes

Because TP/20‐DOI microspheres promoted autophagy and reduced inflammation in diabetic corneas, we next examined whether autophagy activation is functionally required for the therapeutic benefit of TP/20‐DOI microspheres in vivo. When Spautin‐1 was co‐administered, the TP/20‐DOI‐induced acceleration of corneal wound closure at 12, 18, and 24 h post‐injury was substantially reversed (Figure 8a,b), indicating that autophagy is required for efficient re‐epithelialization under this treatment condition.

**FIGURE 8:**
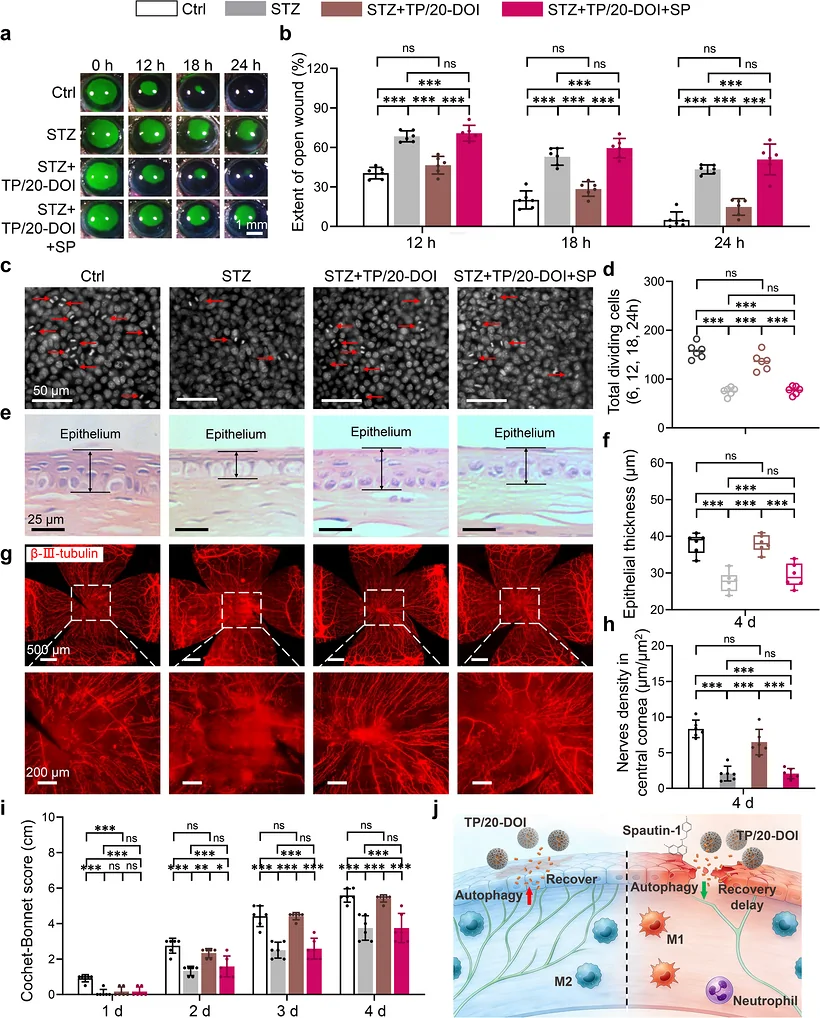
Effects of SP on corneal re‑epithelialization and nerve regeneration in TP/20‐DOI‐treated diabetic mice. (a) Sodium fluorescein staining of corneal wound area from control, STZ, STZ+TP/20‐DOI, STZ+TP/20‐DOI+spautin‐1 group mice. (b) Comparison of corneal wound closure among the four group mice at 12, 18, and 24 h after epithelial abrasion. (c) DAPI staining of corneal epithelial basal cells observed under a 40× objective lens. (d) Difference in the total number of dividing cells across all time points (6, 12, 18, 24 h) after epithelial abrasion among the four group mice. (e) H&E staining of paraffin‐embedded corneal sections at 4 days after epithelial abrasion. (f) Comparison of epithelial thickness among the four group mice. (g) Immunostaining of corneal nerve fibers at 4 days after epithelial abrasion with anti‐β‐III tubulin antibody. (h) Comparison of nerve density in central cornea (1 mm × 1 mm) among the four group mice. (i) Difference in corneal sensitivity in the four group mice at 1, 2, 3, and 4 days following corneal abrasion. (j) Schematic illustration of the effects of SP on wound healing in TP/20‐DOI‐treated diabetic corneas. Data are presented as mean ± SD. Statistical differences were analyzed by one‑way ANOVA followed by Tukey's multiple comparisons test, *n* = 6 mice per group. ns: *p* > 0.05, *: *p* < 0.05, **: *p* < 0.01, ***: *p* < 0.001. SP: spautin‐1; TP/20‐DOI, TSPBA‐PVA loaded 20‐Deoxyingenol; STZ, streptozotocin; DAPI, 4',6‐diamidino‐2‐phenylindole; SD, standard deviation; ns, no significance.

Autophagy inhibition also attenuated the increase in proliferating epithelial basal cells and the restoration of epithelial thickness induced by TP/20‐DOI microspheres (Figure 8c–f), showing that the beneficial effect of TP/20‐DOI microspheres on epithelial reconstruction depends on this pathway. Consistent with this finding, ZO‐1 staining at 24 h after injury showed that TP/20‐DOI increased the ZO‐1‐positive junctional length in the corneal epithelium of STZ mice, whereas co‐administration of spautin‐1 reversed this effect (Figure S6c,d). These data further support that autophagy activation contributes to the restoration of tight‐junction‐associated epithelial integrity during corneal repair.

Consistent with these findings, adoptive transfer of Spautin‐1‐treated BMDMs also attenuated the pro‐healing effect of TP/20‐DOI microspheres, as evidenced by delayed corneal wound closure and reduced numbers of proliferating epithelial cells (Figure S11). This result further supports that macrophage autophagy contributes not only to the anti‐inflammatory action of TP/20‐DOI microspheres, but also to their pro‐repair effect during diabetic corneal wound healing.

Likewise, the improvement in corneal nerve density and corneal sensitivity was significantly impaired by spautin‐1 (Figure 8g–i), indicating that neural recovery is also linked to autophagy activation. Taken together, these results demonstrate that autophagy activation is not simply associated with TP/20‐DOI treatment, but is functionally required for the improvement in epithelial healing and nerve regeneration (Figure 8j). In this way, the molecular changes identified in Figures 6 and 7 are directly connected to the tissue‐level therapeutic outcome.

## 3 Discussion

This study establishes that ROS‐responsive TP/20‐DOI microspheres represent a promising therapeutic strategy for diabetic corneal wound healing. The mechanism revolves around a coordinated sequence: autophagy activation drives a metabolic shift toward oxidative phosphorylation in macrophages, which in turn promotes their polarization toward an anti‐inflammatory, pro‐repair M2 phenotype. This reprogramming at the cellular level translates into accelerated re‐epithelialization, nerve regeneration, and attenuated inflammation in vivo. A key finding is that these benefits are mediated through autophagy‐dependent suppression of macrophage‐derived neutrophil chemoattractants (CXCL3/5), effectively breaking a cycle of pathological neutrophil infiltration that impedes healing. This study thus links fundamental cellular homeostasis (autophagy) to metabolic fitness, immune cell fate, and ultimately functional tissue repair in the diabetic cornea.

The translation of this mechanistic insight into an effective therapy critically depended on overcoming the formidable drug‐delivery barriers of the ocular surface. To this end, we repurposed the TP hydrogel—a ROS‐responsive network formed through dynamic boronic ester bonds between TSPBA and PVA [50]—into an ocular therapeutic platform. Although TP‐based systems have been successfully used to modulate redox‐active microenvironments in extraocular tissues, such as bone and skin [51, 52, 53], their application in ophthalmology remained unexplored. Here, we engineered this smart biomaterial into patient‐friendly, microsphere‐based eye drops optimized for ocular surface delivery. This formulation leverages the intrinsic ROS‐scavenging capacity of TP to simultaneously quench pathological oxidative stress and enable on‐demand, sustained release of the encapsulated therapeutic agent. By doing so, it addresses key limitations of conventional eye drops, particularly poor corneal retention and low bioavailability [23, 24, 25], thereby establishing a targeted delivery strategy for diabetic keratopathy.

Recent studies have demonstrated the therapeutic potential of ROS‐responsive ocular delivery systems in several ocular‐surface diseases, including fungal keratitis, dry eye disease, and corneal neovascularization. In these reported systems, the main formulation advances included enhanced corneal penetration, prolonged local retention, oxidative‐stress‐responsive drug release, and combined antioxidant and anti‐inflammatory activity [54, 55, 56]. By contrast, the present platform was designed for the sterile inflammatory microenvironment of diabetic corneal injury, where the goal is not only to improve ocular residence but also to generate a localized autophagy‐activating signal that reshapes macrophage immunometabolism. Thus, the distinctive value of TP/20‐DOI microspheres lies not simply in ROS responsiveness itself, but in coupling ROS‐responsive topical delivery with macrophage‐centered immunometabolic repair.

In parallel, our work advances the therapeutic potential of the encapsulated agent itself, 20‐DOI. While 20‐DOI has previously been recognized for its cytoprotective and autophagy‐inducing properties in other disease contexts [19, 20, 22], its role in diabetic corneal repair had remained unexplored. Here, we demonstrate that 20‐DOI potently enhances corneal wound healing and, importantly, provide evidence that it activates autophagy in macrophages under high‐glucose conditions—a key cell type in corneal immune homeostasis. To leverage this mechanism, we integrated 20‐DOI into the aforementioned ROS‐responsive TP microsphere system. This combined platform concentrates 20‐DOI release at the wound site to generate a localized and sustained autophagy‐activating signal. Thus, our work not only expands the biological repertoire of 20‐DOI but also, through this smart formulation, underscores a central tenet of modern pharmacotherapy: controlled, targeted delivery is as critical as the therapeutic agent itself. Importantly, previous studies in diabetic corneal repair have shown that autophagy activation can promote epithelial healing and nerve regeneration, for example through miR‐542‐3p inhibition [13] or lncRNA GTF3C1‐mediated regulation [57], but these studies did not employ a ROS‐responsive topical carrier and did not connect autophagy activation to macrophage metabolic polarization.

A pivotal finding of our study vividly demonstrates the principle of targeted delivery: TP/20‐DOI microspheres are markedly superior to free 20‐DOI in vivo, despite showing largely similar effects in vitro on macrophage polarization and metabolism. This apparent discrepancy likely stems from key differences between simplified cell culture systems and the complex ocular surface microenvironment. In vitro, drugs are directly accessible to cells, bypassing dynamic ocular surface barriers such as tear film clearance and the multi‐layered cornea. In contrast, the ROS‐responsive TP microspheres provide sustained, targeted drug release at the pathological site, an advantage amplified by the material's bioadhesion. Thus, the in vivo efficacy gap underscores that overcoming physiological barriers through intelligent delivery is paramount for therapeutic success. This interpretation is further supported by our ocular‐retention experiment, in which the microsphere formulation showed prolonged corneal‐surface fluorescence compared with free 20‐DOI, consistent with enhanced local residence on the ocular surface.

Importantly, the therapeutic performance of TP/20‐DOI microspheres was further benchmarked against bFGF, which was included as a clinically relevant reference treatment because of its documented pro‐reparative effects on the corneal epithelium and its reported benefit in corneal sensory recovery [45, 46, 47]. Within our diabetic corneal injury model, TP/20‐DOI microspheres showed greater efficacy than bFGF in accelerating corneal epithelial wound closure, whereas no significant difference was observed between the two groups in terms of corneal nerve repair (Figure S8). These data indicate that the therapeutic advantage of TP/20‐DOI microspheres is most evident in epithelial resurfacing, while its effect on nerve repair should be interpreted as comparable rather than superior to bFGF under the present experimental conditions. This pattern is mechanistically meaningful, because bFGF primarily acts as a trophic pro‐reparative factor, whereas TP/20‐DOI microspheres combine ROS‐responsive local delivery with sustained autophagy activation and macrophage immunometabolic reprogramming, thereby targeting both corneal repair and the inflammatory microenvironment that constrains healing in diabetic corneal injury. Accordingly, the therapeutic significance of TP/20‐DOI microspheres lies not only in its superiority over free 20‐DOI in vivo, but also in its favorable performance relative to a clinically relevant reference therapy, particularly with respect to epithelial wound closure.

Having established the delivery‐dependent efficacy of the platform, we next delineated its coherent mechanism of action. Delving into the mechanistic hierarchy, our data construct a coherent “autophagy‐metabolism‐polarization” axis. The robust activation of autophagy by 20‐DOI, congruent with its documented role [19, 58] and critically relevant given the autophagy impairment in diabetic corneas [13, 14], serves as the initiation point. Beyond its role in bulk cellular clearance, activated autophagy promotes the removal of damaged mitochondria through mitophagy [38, 39], thereby facilitating a metabolic reprogramming from glycolysis to oxidative phosphorylation (OXPHOS). Such metabolic rewiring is mechanistically meaningful rather than merely correlative, because pro‐inflammatory macrophage (M1) activation is typically associated with enhanced glycolysis, whereas reparative macrophage (M2) states are preferentially supported by mitochondrial oxidative metabolism [59, 60, 61]. Accordingly, the coordinated OCR/ECAR, metabolite, and enzyme changes observed in Figure 4 suggest that TP/20‐DOI microspheres restore a bioenergetic state that is permissive for reparative macrophage function rather than sustained inflammatory activation. Thus, our study empirically connects these dots: TP/20‐DOI‐induced autophagy begets an OXPHOS‐dominant metabolic state, which then licenses and sustains the M2 polarization program.

This intracellular reprogramming directly shapes the extracellular immune microenvironment. We identify a precise effector mechanism: autophagy‐modulated, M2‐polarized macrophages markedly downregulate the secretion of specific neutrophil chemokines, CXCL3 and CXCL5. This establishes a direct link between macrophage intrinsic autophagy and the control of neutrophil recruitment, a cell type notoriously involved in tissue damage during diabetic healing [15]. The reversal of this chemokine suppression and the consequent resurgence of neutrophil influx upon autophagy inhibition solidify the causality of this “autophagy‐macrophage‐chemokine‐neutrophil” axis. Consequently, the therapeutic action of TP/20‐DOI microspheres can be visualized as a two‐pronged strategy: it simultaneously reprograms macrophage function from within and dampens their ability to summon damaging neutrophils from without, thereby resolving inflammation and creating a permissive environment for epithelial and neural restoration. This mechanism also distinguishes our work from earlier macrophage‐polarization‐based strategies in diabetic corneal repair. For example, BM‐MSC/TSG‐6 treatment was reported to accelerate diabetic corneal epithelial wound healing in association with a macrophage switch toward an anti‐inflammatory phenotype [62]. Our study extends that line of work by identifying an upstream autophagy‐metabolism‐polarization program in macrophages and by implementing it through a topical biomaterial formulation rather than through a cell‐based intervention. From a translational standpoint, this acellular, microsphere‐based eye‐drop formulation may also offer practical advantages over live‐cell therapies, including simpler dose standardization, easier storage and administration, and fewer manufacturing and quality‐control complexities. By contrast, clinical translation of MSC‐based products is well known to face challenges related to product heterogeneity, reproducibility, and release testing [63, 64].

We acknowledge certain limitations that frame the interpretation of our study and guide future work. First, although the STZ‐induced type 1 diabetes model is widely used in diabetic corneal research and was appropriate for proof‐of‐concept evaluation, it does not reproduce the obesity‐ and insulin‐resistance‐associated metabolic context of type 2 diabetes. Future studies should therefore extend validation to complementary models such as db/db mice and diet‐induced obesity/high‐fat‐diet models, which may better capture additional dimensions of clinical diabetic keratopathy. Second, while our study demonstrated beneficial effects during the acute healing phase, longer‐term follow‐up will be necessary to more fully assess the durability of therapeutic efficacy and the long‐term biosafety of TP/20‐DOI microspheres. Third, although Spautin‐1 inhibition strongly supports a central role for autophagy, more specific genetic approaches will be valuable for refining pathway causality. Finally, detailed in vivo pharmacokinetic tracking of the microspheres would precisely map drug exposure to the observed pharmacodynamic effects.

## 4 Conclusion

In conclusion, this study establishes ROS‐responsive TP/20‐DOI microspheres as a biomaterial‐based therapeutic platform for diabetic corneal wound healing. Mechanistically, our findings support an autophagy–metabolism–polarization axis in which 20‐DOI‐driven autophagy activation promotes oxidative metabolic reprogramming in macrophages, facilitates anti‐inflammatory polarization, and contributes to inflammatory resolution and tissue repair. Functionally, incorporation of 20‐DOI into the TP microsphere system improves its local therapeutic performance on the ocular surface. These results provide mechanistic insight and preclinical support for TP/20‐DOI microspheres as a potential topical strategy for diabetic keratopathy, while also informing the design of ROS‐responsive therapies for other oxidative stress‐associated defects in tissue healing.

## 5 Materials and Methods

### 5.1 Fabrication of Microfluidic Chips and TP/20‐DOI Microspheres

#### 5.1.1 Microfluidic Chip Design

A commercially available microfluidic chip and associated fluid‐handling system (MesoBioSystem) were used. The chip comprises a glass substrate with two inlets (for oil and water phases) and one outlet (channel depth: 100 µm), connected to a silicone tube for microsphere collection.

#### 5.1.2 Preparation of Microspheres

The oil‐phase consisted of mineral oil containing 10% (v/v) Span 80, and the aqueous phase consisted of 5% (w/v) poly (vinyl alcohol) (PVA) containing 20‐DOI. The oil phase and aqueous phase were infused into the respective inlets of the microfluidic chip. By adjusting the relative pressure of the aqueous phase, water‐in‐oil droplets were generated at the junction and collected from the outlet. The collected droplets were kept at 4°C for 12 h, centrifuged at 5000 rpm for 5 min to remove the upper oil phase, and then resuspended in 1 mL phosphate buffered saline (PBS) (pH 7.4). TSPBA was dissolved in 0.1 M borate buffer (30 mg/mL, pH 9) and mixed with the PVA microspheres, followed by gentle stirring at 37°C for 12 h to allow sufficient TSPBA‐PVA crosslinking. The resulting microspheres were collected by repeated centrifugation at 3000 rpm and washed with PBS. Examined under an optical microscope, and stored at 4°C. Unless otherwise specified, unloaded carrier microspheres are referred to as TP microspheres, whereas 20‐DOI‐loaded microspheres are referred to as TP/20‐DOI microspheres throughout the manuscript.

### 5.2 Microstructural Characterization and Degradation Analysis of TP and TP/20‐DOI Microspheres

For microstructural characterization analysis, TP and TP/20‐DOI microspheres were air‐dried, sputter‐coated with gold (10 nm), and imaged using a Hitachi N7000 scanning electron microscope. Particle size distribution was analyzed with ImageJ software.

For degradation analysis, TP and TP/20‐DOI microspheres were incubated in PBS containing different concentrations of H_2_O_2_ at 37°C for 72 h. After incubation, the remaining microspheres were collected by centrifugation and weighed after removal of excess liquid. The degradation rate (%) was calculated according to the following formula: Degradation rate (%) = [(W_0_ − Wt)/W_0_] × 100, where W_0_ represents the initial microsphere weight and Wt represents the residual weight after incubation.

### 5.3 Determination of Drug‐Loading Content and Encapsulation Efficiency

The drug‐loading (DL) content and encapsulation efficiency (EE) of 20‐DOI in TP/20‐DOI microspheres were determined to evaluate the relationship between the initial feeding concentration and the final drug content in the microspheres. Briefly, precursor aqueous solutions containing different initial concentrations of 20‐DOI were prepared, and TP/20‐DOI microspheres were fabricated as described above. The resulting microspheres were then dissolved in 1 mM H_2_O_2_ buffer to ensure complete degradation. After centrifugation at 500 × g for 5 min, the supernatants were collected and subjected to HPLC analysis under validated analytical conditions. The DL and EE were calculated according to the following equations: DL (%) = (actual amount of 20‐DOI in the microspheres / total weight of dried TP/20‐DOI microspheres) × 100; EE (%) = (actual amount of 20‐DOI in the microspheres / theoretical amount of 20‐DOI initially added) × 100.

### 5.4 Evaluation of Ocular Formulation Properties

The viscosity of the TP/20‐DOI microsphere suspension was measured using a MARS III rheometer (HAAKE, Germany) over a shear rate range of 0.1‐100 s^−^^1^ at 25°C. Sedimentation behavior was evaluated using a dispersion/stability analyzer (LUMiSizer, Model LS 611). The sedimentation ratio curve was generated using the integration module of the LUMiSizer, which calculated the integral values of the original transmittance data at each time point over 24 h.

The redispersibility of the TP/20‐DOI microsphere suspension was assessed by gently rotating the container through 180 degrees. The number of rotations required to obtain a visually homogeneous suspension without obvious precipitation was recorded. After redispersion, samples were collected from the upper, middle, and lower layers of the suspension, and the 20‐DOI content was determined by HPLC to verify dose uniformity.

Mucoadhesive properties were evaluated using a TA.XT plus Texture Analyzer (Stable Micro Systems, UK), and the results were expressed as the work of adhesion. Wash‐off resistance was examined ex vivo using ocular tissue under continuous rinsing with simulated tear fluid, and the time required for complete removal of the microspheres from the mucosal surface was recorded.

### 5.5 HCEC Cytotoxicity Assay

HCECs were used to assess the cytocompatibility of 20‐DOI in vitro. Cells were seeded into 96‐well plates at a density of 5 × 10^3^ cells per well and exposed to a series of 20‐DOI concentrations for 24 h. Cell viability was then assessed using a CCK‐8 assay (Glpbio; Cat. No. GK10001) according to the manufacturer's instructions. Absorbance at 450 nm (OD_450_) was measured using a microplate reader. Cell viability was calculated using the following formula: Cell viability (%) = [(Ae – Ab) / (Ac – Ab)] × 100%, where Ae is the absorbance of the experimental well containing cells, culture medium, and the indicated concentration of 20‐DOI; Ab is the absorbance of the blank well containing culture medium only without cells; and Ac is the absorbance of the control well containing cells and culture medium but no 20‐DOI. The maximum non‐toxic concentration was defined as the highest concentration that did not noticeably reduce cell viability compared with the control group.

### 5.6 Drug Release Profiling

The release of 20‐DOI from TP microspheres were evaluated in simulated body fluid (SBF; Leagene, Beijing, China; CZ0403) with or without 1 mM H_2_O_2_. Microspheres were incubated in 10 mL of release medium at 37°C. Aliquots (100 µL) were collected every 6 h for 30 h and replaced with fresh medium. 20‐DOI concentration was quantified by mass spectrometry. The released amount was calculated as C  =  D/(V × t), where C is concentration, D is total drug amount, V is flow rate, and t is detection time. The cumulative release percentage was calculated using the following equation: Cumulative release (%) = (C_t_/C_0_) × 100, where C_t_ represents the cumulative amount of 20‐DOI released at time t, and C_0_ represents the total amount of loaded 20‐DOI. The released concentration for each 6‐h interval was calculated as: C_interval_ (mg/mL) = C_t_ – C_t‐1_, where C_t‐1_ represents the amount of 20‐DOI concentration measured at the last 6 h interval, except for 0 h.

### 5.7 RAW264.7 Cell Culture

RAW264.7 cells were maintained in DMEM (Gibco, Thermo Fisher Scientific, Waltham, MA, USA; Cat. No. 11995073) supplemented with 10% (v/v) fetal bovine serum (Gibco, Cat. No. 10500064) at 37°C in a humidified atmosphere containing 5% CO_2_. To establish a high‐glucose (HG) model, cells were cultured in medium containing 35 mM glucose for 48 h. Subsequently, the HG‐treated cells were randomly divided into seven groups: HG control; IL‐10 (20 ng mL^−1^); 20‐DOI (50 µg mL^−1^); TP (TP microspheres prepared from 1 mL PVA raw material); TP/20‐DOI (TP microspheres prepared from 1 mL PVA raw material, containing 1 mg mL^−1^ 20‐DOI); TP/20‐DOI + Spautin‐1 (SP, 0.15 µg mL^−1^; MedChemExpress; Cat. No. HY‐12990); and TP/20‐DOI + Rotenone (Rot, 0.5 µM; Sigma–Aldrich St. Louis, MO, USA; Cat. No. R8875). For the combination group, SP/Rot was added 30 min after TP/20‐DOI treatment. After 24 h of drug exposure, cells were harvested for subsequent analysis.

### 5.8 Enzyme‑Linked Immunosorbent Assay (ELISA)

ELISA was performed according to the manufacturer's protocol (TGF‐β ELISA Kit, Thermo Fisher Scientific, Waltham, MA, USA; Cat. No. KGTGFBE; iNOS ELISA Kit, mlbio, Shanghai, China; Cat. No. ml001883; ARG1 ELISA Kit, Jonlnbio, Shanghai, China; Cat. No. JL13668; CCL22 ELISA Kit, Henghuibio; Cat. No. RD‐RX28161; ITGα4 ELISA Kit, Share‐bio, Shanghai, China; Cat. No. SB‐EM4455). In brief, plates were coated with capture antibody overnight at 4°C, incubated with samples or standards for 2 h at 37°C, followed by detection antibody (2 h) and streptavidin‐HRP (20 min). Color was developed using a 1:1 mixture of H_2_O_2_ and tetramethylbenzidine, absorbance was read at 450 nm, and sample concentrations were determined from a standard curve.

### 5.9 Mechanistic Rescue Experiments for CXCL3/CXCL5 Regulation

RAW264.7 macrophages were cultured under high‐glucose conditions and treated with TP/20‐DOI microspheres as described above. To investigate the involvement of NF‐κB signaling in CXCL3/CXCL5 regulation, the cells were divided into three groups: HG, HG + TP/20‐DOI, and HG + TP/20‐DOI + TNF‐α. Recombinant TNF‐α was administered at a final concentration of 10 ng/mL for 1 h to reactivate NF‐κB signaling. After treatment, cell lysates were prepared using lysis buffer supplemented with protease and phosphatase inhibitors. The levels of phosphorylated NF‐κB p65 and total NF‐κB p65 were determined using Phospho‐NF‐κB p65 and Total NF‐κB p65 ELISA kits (Abcam, UK), and NF‐κB activation was expressed as the ratio of phosphorylated p65 to total p65. The concentrations of CXCL3/5, IL‐10, and IL‐1β in the corresponding culture supernatants were measured by ELISA.

### 5.10 Analysis of Glucose Metabolism

After 3 days of culture, metabolites and enzyme activities were assessed. ATP, lactate, and pyruvate levels were determined using commercial kits (ATP Assay Kit, Beyotime, Shanghai, China; Cat. No. S0026; Lactate Assay Kit, Dojindo, Kumamoto, Japan; Cat. No. L256; Pyruvate Assay Kit, Elabscience, Wuhan, China; Cat. No. E‐BC‐K130‐M). Activities of PFK‐1, PK, HK, LDH, and PDH were measured with corresponding assay kits (PFK‐1 Activity Assay Kit, Saint‐bio, Shanghai, China; Cat. No. BA1194; PK Activity Assay Kit, Saint‐bio; Cat. No. BA1061; HK Activity Assay Kit, Saint‐bio; Cat. No. BA2147; LDH Activity Assay Kit, Saint‐bio; Cat. No. BA1260; PDH Activity Assay Kit, Saint‐bio; Cat. No. BA1059).

### 5.11 RNA Sequencing

RNA was extracted from 20‐DOI‐ and TP‐treated cells using a column‑based method. Quality and concentration were verified with a Nano spectrophotometer. Libraries were sequenced on an Illumina HiSeq 3000 platform. Raw reads were quality‐controlled, and differentially expressed genes (DEGs) were identified with DESeq2 and edgeR (FDR < 0.05, |fold‐change| ≥ 2). Enrichment analyses of GO terms and KEGG pathways were performed on the DEG sets.

### 5.12 Seahorse Extracellular Flux Assay

Cellular metabolism was assessed using a Seahorse XF96 analyzer (Agilent Technologies). Cells were pretreated for 72 h, seeded onto Cell‐Tak‐coated XF96 plates (2 × 10^5^ cells/well), and equilibrated in glucose‐free medium for ECAR measurements. Sequential injections of 10 mM glucose, 1 µM oligomycin, and 50 mM 2‐DG were performed to determine glycolytic function and capacity.

### 5.13 HCEC Scratch Assay

HCECs were seeded into 6‐well plates at a density of 2 × 10^5^ cells per well and cultured until reaching complete confluence. A linear scratch was created in the cell monolayer using a 200 µL pipette tip. Cells were assigned to four groups: HG, HG + 20‐DOI, HG + TP microspheres, and HG + TP/20‐DOI microspheres. Images were captured at 0, 12, and 24 h after scratching, and wound‐closure percentage was quantified using ImageJ software.

### 5.14 Ocular‐Surface Retention Assay

Cy5‐labeled 20‐DOI (MedChemExpress) was formulated either as free eye drops or as TP/20‐DOI microsphere eye drops. After topical administration to mice, corneal fluorescence images and fluorescence intensity were acquired at 0, 3, 6, and 9 h using Bruker MI SE.

### 5.15 Animals and Induction of Diabetes

Male C57BL/6 mice (7–8 weeks old) without ocular diseases were obtained from the Guangdong Medical Laboratory Animal Center and maintained under specific pathogen‐free (SPF) conditions. All animals were housed in a standard environment with free access to food and water. All experimental procedures were conducted in strict accordance with the guidelines approved by the Experimental Animal Committee of Jinan University and the Statement for the Use of Animals in Ophthalmic and Vision Research.

Mice were randomly assigned to a control group or a streptozotocin (STZ)‐induced diabetic group. Diabetes was induced by a single intraperitoneal injection of STZ (200 mg kg^−1^, dissolved in freshly prepared citrate buffer; Sigma–Aldrich, St. Louis, MO, USA; Cat. No. S0130; 0.2 mL per mouse) as described previously [15, 65, 66, 67]. Age‐matched control mice received an equal volume of citrate buffer. One week after injection, blood glucose levels were measured from the tail vein. Mice with two consecutive random blood glucose readings ≥ 16.7 mmol L^−1^ were considered diabetic. Subsequent experiments were performed four weeks after successful model induction. A schematic illustration of the entire animal experiment process was added in Figure S4. Mice were anesthetized with 2% isoflurane inhalation and euthanized by a combination of CO_2_ overdose and cervical dislocation.

### 5.16 Corneal Wound Healing Model

The corneal wound healing model was established according to established protocols described in our previous studies [15, 17, 68, 69, 70, 71]. After anesthesia, a circular area (2 mm in diameter) was demarcated in the central cornea using a trephine under a stereomicroscope. The corneal epithelium within the marked region was then mechanically removed using a golf‐club‐shaped scraper.

### 5.17 Measurement of Reactive Oxygen Species (ROS) Levels in Corneal Cells

Following euthanasia, corneal tissues were excised, minced, and digested into single‑cell suspensions using 4 mg/mL collagenase I (Sigma–Aldrich, St. Louis, MO, USA; Cat. No. C0130) in a 37°C water bath. Cells were washed twice with PBS, collected, and then assayed for intracellular ROS levels using a Reactive Oxygen Species Assay Kit (Beyotime; Cat. No. S0033S) according to the manufacturer's instructions.

### 5.18 Pharmacological Agent Administration in Mice

According to the treatment regimen, mice were divided into the following groups: Control, STZ, STZ + 20‐DOI, STZ + TP, STZ + TP/20‐DOI, and STZ + TP/20‐DOI + Spautin‐1 (SP). Free 20‐DOI eye drops were prepared at a concentration of 1 mg mL^−1^. The TP/20‐DOI microsphere eye‐drop formulation contained 20‐DOI at 1 mg mL^−1^, and the microsphere concentration in the suspension was 2 × 10^4^ microspheres µL^−1^. Thus, the free 20‐DOI and TP/20‐DOI groups were matched with respect to 20‐DOI content. The TP‐alone group received a microsphere suspension at the same particle concentration as the TP/20‐DOI group (2 × 10^4^ microspheres µL^−1^). Spautin‐1 (SP; MedChemExpress; Cat. No. HY‐12990) was dissolved at 1 mg mL^−1^ in a vehicle consisting of 2% (v/v) dimethyl sulfoxide (DMSO), 30% (v/v) polyethylene glycol (PEG) 400, 2% (v/v) Tween‐80, and 66% (v/v) normal saline. As a common drug control, bFGF (4200 IU/mL; Essex Bio‐Pharmaceutical Co., Ltd., Zhuhai, China) was topically administered. All drugs were administered topically via eye drops (4 µL per eye, every 6 h). For the combination treatment group (STZ + TP/20‐DOI + SP), TP/20‐DOI and SP eye drops were administered with a 30‐min interval, with TP/20‐DOI applied first followed by SP. This administration sequence was maintained consistently throughout the treatment period.

### 5.19 Corneal Transmittance Measurement

Corneal optical transmittance at 450 nm was determined using a microplate reader. Corneas were positioned centrally in wells of a 96‐well plate, and absorbance was measured at 450 nm. Blank wells were included for background subtraction, and the corrected absorbance was calculated as the absorbance of the cornea‐containing well minus that of the blank well. The transmittance of each cornea was calculated using the equation T = 10^‐A. For presentation as percentage transmittance, values were converted using T (%) = 10^‐A × 100.

### 5.20 Immunostaining

#### 5.20.1 Immunofluorescence Staining of RAW264.7 Cells

Cells were seeded on coverslips and treated under the indicated conditions. After treatment, cells were fixed with 4% paraformaldehyde in PBS for 15 min, permeabilized with 0.1% Triton X‐100 in PBS for 10 min, and blocked with 5% bovine serum albumin (BSA) in PBS for 15 min at room temperature. Anti‐CD86 (1:1000; Sigma–Aldrich; Cat. No. SAB4700560) and anti‐CD206 (1:1000; Sigma–Aldrich; Cat. No. SAB5700929) antibodies were incubated overnight at 4°C, followed by PE‑conjugated goat anti‐rat IgG antibody (1:500; BioLegend, San Diego, CA, USA; Cat. No.  405406) and fluorescein isothiocyanate (FITC)‐conjugated Donkey anti‐rabbit IgG antibody (1:500; BioLegend; Cat. No.  406403), separately. Nuclei were counterstained with DAPI. Coverslips were mounted using anti‑fade mounting medium.

#### 5.20.2 Immunofluorescence Staining of Corneas

Following euthanasia, whole eyeballs were fixed and cut to preserve the intact cornea with the limbus. Corneal tissues were blocked in 2% BSA solution for 15 min, permeabilized in 0.2% Triton X‐100 prepared in 2% BSA for 15 min, and subsequently incubated with primary antibodies at 4°C overnight. The primary antibodies used were as follows: anti‐β‐III tubulin NL557 (1:40; R&D Systems; Cat. No. NL1195R), anti‐Mouse Ly6G FITC (1:50; BD Biosciences, San Jose, CA, USA; Cat. No. 551460), anti‐Mouse F4/80 PE (1:50; eBioscience, San Diego, CA, USA; Cat. No. 12‐4801‐82), anti‐mouse ZO‐1 CoraLite488 (1:50; Proteintech, Rosemont, IL, USA; Cat. No. CL488‐21773), and anti‐mouse α‐SMA (1:50; Selleck, Houston, TX, USA; Cat. No. F0017), anti‐CXCL3 (1:50; Boster Biological Technology, Wuhan, China; Cat. No. PB0994), and anti‑Mouse CXCL5 (1:50; Proteintech, Chicago, IL, USA; Cat. No. 98417‐2‐RR). For staining requiring a secondary antibody, corneas were washed three times with PBS (10 min each) after primary antibody incubation and then transferred to a solution containing the secondary antibody (Alexa Fluor 488 Donkey anti‐rabbit IgG, 1:50; BioLegend; Cat. No. 406416) for overnight incubation at 4°C in the dark. After three additional PBS washes, the corneas were cut into four petals and flat‐mounted on glass slides with an anti‐fade mounting medium containing DAPI.

### 5.21 Corneal Wound Healing Assessment

#### 5.21.1 Wound Closure

Wound area was stained by fluorescein sodium at 0, 12, 18, and 24 h post‐injury and photographed under a stereomicroscope. The fluorescein‐positive area at each time point was quantified using ImageJ software. The percentage of the remaining wound area relative to the initial wound area was calculated to assess the rate of corneal epithelial wound repair.

#### 5.21.2 Epithelial Cell Proliferation

The cornea was divided into five zones: Zone 1 (limbus), Zone 2 (peripheral cornea), and Zones 3–5 (the epithelial wound area; see Figure S12). Proliferating epithelial cells along the basal layer were observed under a 40× objective lens. The total number of such cells within 17 microscopic fields of view, counted along two perpendicular central axes crossing the corneal center, was determined and defined as the number of proliferating epithelial cells.

#### 5.21.3 Epithelial Thickness

Mouse eyeballs were fixed in 4% paraformaldehyde for 24 h, embedded in paraffin, and longitudinally sectioned along the axis connecting the optic nerve and corneal center. The sections were stained with hematoxylin and eosin (H&E), and the thickness of the central corneal epithelium was measured using Olympus cellSens software.

#### 5.21.4 Nerve Fiber Regeneration

Corneal nerve fibers were imaged using a DeltaVision microscope imaging system. The nerve pixel area within the central corneal region (1 mm × 1 mm) was analyzed using ImageJ software. The total nerve fiber length was measured, and the nerve density in the central cornea was calculated by dividing the total length by the central corneal area.

#### 5.21.5 Corneal Sensitivity

Corneal sensitivity was evaluated on days 1–4 post‑injury using a Cochet–Bonnet aesthesiometer. The tactile threshold at the central cornea was measured daily at a fixed time point. The shortest length of the nylon filament that elicited a blink reflex was recorded and used to assess the recovery of corneal nerve function.

### 5.22 Analysis of Neutrophil Recruitment to the Wound Area

Under a 40× objective lens, four quadrants within corneal Zone 3 were examined. One field of view was randomly selected per quadrant for imaging (Figure S12). Neutrophils within each field were counted, and the average count from the four fields represented the extent of neutrophil recruitment to the wound area.

### 5.23 Adoptive Transfer of BMDMs

F4/80^+^ BMDMs were sorted using a BD FACSAria II cell sorter and divided into two groups for treatment with either Spautin‐1 (0.15 µg mL^−1^, 3 h) or vehicle. For tracing, cells were labeled with CellTracker Green CMFDA Dye (Thermo Fisher Scientific, Waltham, MA, USA; Cat. No. C2925) before transfer. Diabetic recipient mice received tail vein injections of BMDMs at 0 and 12 h after corneal epithelial injury (1 × 10^6^ cells per mouse per injection). The presence of transferred BMDMs in the injured cornea was confirmed by fluorescence tracing. Neutrophil infiltration, wound closure and epithelial cell proliferation were evaluated as described above.

### 5.24 Immunofluorescence Quantification of CXCL3 and CXCL5 Expression

Images of the chemokines CXCL3 and CXCL5 were captured using a 40× objective lens on a fluorescence microscope. Seventeen fields of view along two perpendicular central axes crossing the corneal center were photographed for each sample (Figure S12). The fluorescence intensity of the chemokines was subsequently quantified using ImageJ software.

### 5.25 RNA Extraction

For RNA extraction from corneal tissues, the tissue was minced and homogenized in 1 mL of Buffer RZ lysis buffer (Tiangen, Beijing, China; Cat. No. RK145) using a handheld homogenizer (Qiagen, Germantown, MD, USA). For RNA extraction from cells, the cell pellet was directly lysed in 0.5–1 mL of Buffer RZ. Total RNA was then isolated using the Tiangen Total RNA Kit (Tiangen, Beijing, China; Cat. No. DP419) according to the manufacturer's protocol. RNA concentration was measured with a NanoDrop spectrophotometer, and RNA integrity was assessed by agarose gel electrophoresis.

### 5.26 Reverse Transcription Quantitative PCR (RT‐qPCR)

mRNA was reverse‐transcribed into cDNA using the Toyobo Reverse Transcription Kit (Toyobo, Osaka, Japan; Cat. No. FSQ‐101). The resulting cDNA was used as a template for quantitative PCR amplification with THUNDERBIRD SYBR qPCR Mix (Toyobo, Osaka, Japan; Cat. No. QPS‐201) on a real‐time PCR system. *Gapdh* was used as the reference gene for normalization. The relative expression levels of target genes were calculated using the 2^−ΔΔCt^ method. Primer sequences used in this study are listed in Table S3.

### 5.27 Assessment of Autophagy Levels

Corneal tissues from mice were thoroughly minced and digested into single‐cell suspensions using 4 mg/mL collagenase I in a 37°C water bath. After two washes with PBS, the cells were collected. Autophagy levels were then assessed using an autophagy staining detection kit (Beyotime; Cat. No. C3018S) according to the manufacturer's instructions.

### 5.28 Statistical Analysis

Data are expressed as mean ± standard deviation (SD) at least three independent experiments. Comparisons between two groups were made using unpaired Student's t‐test; multiple groups were compared by one‐way ANOVA with Tukey's post hoc test. Analyses were conducted in GraphPad Prism 10.0. Significance levels are denoted as *p < 0.05, **p < 0.01, and ***p < 0.001; “ns” indicates not significant.

## Author Contributions

**Ying Jiao**: methodology, software, data curation, investigation, validation, formal analysis. **Haijiang Qiu**: methodology, software, data curation, investigation, validation, formal analysis. **Zhudan Zhuang**: methodology, software, validation. **Jingbin Xie**: methodology, software. **Hanxiao Sun**: methodology, software. **Huifei Lan**: methodology, software. **Xing Hua**: methodology, software, validation. **Wanying Chen**: methodology, software, data curation, investigation, validation, formal analysis. **Jiaxin Wu**: methodology, software, data curation, investigation, validation, formal analysis. **Yuheng Liu**: methodology, software. **Weihan Zheng**: methodology, software, validation, investigation, formal analysis, data curation. **Xin Sun**: methodology, software. **Yunxia Xue**: methodology, software, validation, supervision. **Zhijie Li**: conceptualization, funding acquisition, resources, writing – review and editing. **Jun Liu**: conceptualization, investigation, funding acquisition, writing – original draft, writing – review and editing, project administration, resources. **Ting Fu**: methodology, software, validation, supervision. **Guobing Chen**: conceptualization, funding acquisition, writing – review and editing, resources. **Zhaojin Li**: methodology, software. **Shiyu Li**: conceptualization, investigation, funding acquisition, project administration, resources, writing – original draft, writing – review and editing. **Jingheng Du**: methodology, software, validation. **Jingyi Gu**: methodology, software, validation.

## Conflicts of Interest

The authors declare no conflicts of interest.

## Supporting information




**Supporting File**: advs77232‐sup‐0001‐SuppMat.docx.

## Data Availability

The data that support the findings of this study are available from the corresponding author upon reasonable request.
