# Supplementary material for: ROS‐Responsive Eye Drop for Controlled Release of 20‐Deoxyingenol Improves Diabetic Corneal Healing by Activating Macrophage Autophagy and Metabolic Reprogramming
Source: Adv Sci (Weinh). 2026 Aug 18:e77232. Online ahead of print. doi: 10.1002/advs.77232 (PMC13482942; doi:10.1002/advs.77232)
Supplement: Supplementary file 1 [file ADVS-9999-e77232-s001.docx]

**1 Ocular formulation performance of TP/20-DOI microspheres**

The ocular formulation properties of the TP/20-DOI microsphere suspension were further evaluated to determine its suitability for topical ophthalmic administration. The suspension exhibited typical shear-thinning behavior, with viscosity progressively decreasing as the shear rate increased from 0.1 to 100 s⁻¹ (**Figure S1a**). Its physical stability was further assessed using a LUMiSizer dispersion analyzer. As shown in **Figure S1b**, the integral transmission increased rapidly at the initial stage and then gradually reached a plateau during prolonged centrifugation, indicating that sedimentation mainly occurred early without further continuous phase separation. No obvious irreversible sedimentation or aggregation was observed throughout the accelerated sedimentation test. In addition, the suspension could be completely redispersed after 7 ± 1 inversions without visible caking. Comparable drug contents in the upper, middle, and lower layers further confirmed its satisfactory redispersibility and suspension homogeneity (**Figure S1c**).

**Figure S1**

Ocular formulation performance of TP/20-DOI microspheres. (a) The viscosity-shear rate curve of TP/20-DOI 1 microspheres. (b) Kinetics of sedimentation of TP/20-DOI 1 microspheres. (c) Dose uniformity of TP/20-DOI 1 microspheres. ns: p > 0.05.

**2 *In vitro* Cytotoxicity Evaluation of TP/20-DOI Microspheres**

To assess the biosafety of the formulations, RAW264.7 cells were treated with 20-DOI, TP, or TP/20-DOI for up to 72 hours, and cytotoxicity was evaluated by Calcein AM/PI staining. None of the treatments affected the viability of RAW264.7 cells at 24, 48, or 72 hours (**Figure S2a**, **b**). Cell proliferation assays revealed that all groups—including high glucose (HG), 20-DOI, TP, and TP/20-DOI—exhibited significant proliferation over time. While no differences in viability were observed among the four groups within 24 and 48 hours, the TP/20-DOI group showed a statistically significant increase in proliferative activity at 72 hours compared to the HG, 20-DOI and TP groups (**Figure S2c**). These results indicate that TP/20-DOI displays excellent cytocompatibility and does not compromise macrophage viability.

**Figure S2**

Analysis of the proliferation and viability of RAW264.7 cells. a) Live/dead staining images of RAW264.7 cells at 24, 48 and 72 hours. Scale bars = 100 μm. b) Quantitative analysis of the live/dead ratio of RAW264.7 cells (n = 5). c) Cell viability of RAW264.7 cells assessed by the CCK-8 assay at 24, 48 and 72 hours (n = 5). Data are presented as mean ± SD. Statistical differences were analyzed by one-way ANOVA followed by Tukey's multiple comparisons test. ns: *p* > 0.05, *: *p* < 0.05. HG, high glucose; 20-DOI, 20-Deoxyingenol; TP, TSPBA-PVA; TP/20-DOI, TSPBA-PVA loaded 20-Deoxyingenol; SD, standard deviation; ns, no significance.

**3 Effects of Rotenone on Macrophage Metabolism and Polarization**

Oxygen consumption rate (OCR) analysis showed that, under high-glucose (HG) conditions, TP/20-DOI microspheres increased basal respiration, ATP production, and maximal respiratory capacity, while reducing proton leak in RAW264.7 cells; these effects were abolished by rotenone treatment (**Figure S3a–e**). In parallel, extracellular acidification rate (ECAR) analysis demonstrated that TP/20-DOI microspheres reduced basal glycolysis and glycolytic capacity, whereas these effects were also reversed by rotenone (**Figure S3g**, **h**).

We then examined additional indices of glucose metabolism. Compared with the TP/20-DOI group, the TP/20-DOI + rotenone group showed reduced ATP production (**Figure S3i**), increased activities of the glycolytic enzymes phosphofructokinase-1 (PFK-1), hexokinase (HK), pyruvate kinase (PK), and lactate dehydrogenase (LDH) (**Figure S3j**, **k**), decreased pyruvate dehydrogenase (PDH) activity (**Figure S3k**), increased lactate accumulation, and reduced pyruvic acid levels (**Figure S3l**).

Consistently, rotenone treatment downregulated the mRNA expression of key tricarboxylic acid (TCA) cycle enzymes, including alpha-ketoglutarate dehydrogenase (*α-Kgdh*), isocitrate dehydrogenase (*Idh*), and citrate synthase (*Cs*), as well as oxidative phosphorylation-related subunits, including NADH dehydrogenase [ubiquinone] 1 beta subcomplex subunit 3 (*Ndufb3*), cytochrome c oxidase subunit 3 (*Mtco3*), and succinate dehydrogenase subunit B (*Sdhb*), in TP/20-DOI microsphere-treated RAW264.7 cells (**Figure S3m**, **n**).

In addition, macrophage polarization analysis showed that rotenone decreased ARG1 and IL-10 protein expression but increased iNOS, IL-1β, CXCL3, and CXCL5 protein expression in TP/20-DOI microsphere-treated RAW264.7 cells (**Figure S3o**, **p**). At the transcriptional level, rotenone also increased the expression of M1-associated genes, including *Tnf*, *Il1b*, *Cd86*, and *Nos2* (**Figure S3q**). These findings indicate that inhibition of mitochondrial oxidative metabolism impairs TP/20-DOI microsphere-induced M2-like polarization and instead shifts macrophages toward a more pro-inflammatory phenotype.

**Figure S3**

Effects of Rotenone on the Metabolism and Polarization of TP/20-DOI Microsphere-treated RAW264.7 cells under high-glucose conditions. a-e) OCR parameters measured by Seahorse metabolic analysis (n=3). f-h) ECAR analysis obtained via Seahorse metabolic analysis (n=3). i) ATP production measured at different time points post-treatment (n=3). j) Activities of PFK-1, HK and PK measured at different time points post-treatment (n=3). k) Activities of LDH and PDH (n=3). l) Content of lactate and pyruvate (n=3). m) RT-qPCR analysis of *α-Kgdh*, *Idh* and *Cs* expression (n=3). n) RT-qPCR analysis of *Ndufb3*, *Mtco3* and *Sdhb* expression (n=3). o, p) Protein expression of ARG1, iNOS, CXCL3, CXCL5, IL-10, and IL-1β. q) RT-qPCR analysis of *Tnf*, *Il1b*, *Cd86*, and *Nos2*. Data are presented as mean ± SD. Statistical differences were analyzed by one-way ANOVA followed by Tukey's multiple comparisons test. ns: *p* > 0.05; *: *p* < 0.05; **: *p* < 0.01; ***,†††: *p* < 0.001. *: HG+TP/20-DOI group vs. HG group, †: HG+TP/20-DOI+Rot group vs. HG+TP/20-DOI group. HG, high glucose; TP/20-DOI, TSPBA-PVA loaded 20-Deoxyingenol; Rot, Rotenone; OCR, Oxygen Consumption Rate; ECAR, Extracellular Acidification Rate; PFK-1, phosphofructokinase-1; HK, hexokinase; PK, pyruvate kinase; LDH, lactate dehydrogenase; PDH, pyruvate dehydrogenase; RT-qPCR, reverse transcription quantitative polymerase chain reaction; *α-Kgdh*, alpha-ketoglutarate dehydrogenase; *Idh*, isocitrate dehydrogenase; *Cs*, citrate synthase; *Ndufb3*, NADH dehydrogenase [ubiquinone] 1 beta subcomplex subunit 3; *Mtco3*, cytochrome c oxidase subunit 3; *Sdhb*, succinate dehydrogenase subunit B; ARG1, arginase 1; iNOS, inducible nitric oxide synthase; *Tnf*, tumor necrosis factor; *Il1b*, interleukin 1beta; SD, standard deviation; ns, no significance.

**4 Schematic Illustration of the Animal Experimental Timeline**

On day 1, mice received an intraperitoneal injection of STZ. On day 7, tail-vein blood was collected to measure random blood glucose levels, and mice with glucose levels ≥ 16.7 mmol L^-1^ were considered successfully diabetic. On day 35, diabetic mice were subjected to corneal epithelial injury and then treated topically with 20-DOI, TP, TP/20-DOI, bFGF, or SP. The effects of these treatments on corneal wound healing and inflammatory responses in diabetic mice were subsequently evaluated.

**Figure S4**

A schematic illustration of the experimental timeline for diabetic corneal epithelial injury and topical treatment. STZ, streptozotocin; i.P., intraperitoneal injection; 20-DOI, 20-Deoxyingenol; TP, TSPBA-PVA; TP/20-DOI, TSPBA-PVA loaded 20-Deoxyingenol; bFGF, basic fibroblast growth factor; SP, spautin-1.

**5 Ocular-surface Retention of 20-DOI in Mice**

Cy5-labeled 20-DOI was administered topically either as free 20-DOI eye drops or as TP/20-DOI microsphere eye drops. Corneal fluorescence was recorded at 0, 3, 6, and 9 h after instillation. In both groups, fluorescence intensity gradually decreased over time. However, the free 20-DOI group showed no detectable signal at 6 h, whereas the TP/20-DOI group retained detectable fluorescence until 9 h (**Figure S5a**). Quantitative analysis showed significantly higher fluorescence intensity in the TP/20-DOI group than in the free 20-DOI group at 3 and 6 h (**Figure S5b**), indicating prolonged ocular-surface retention of the TP/20-DOI formulation.

**Figure S5**

Comparison of the ocular-surface retention time of 20-DOI between the free 20-DOI group and the TP/20-DOI group in STZ-treated mice. a) Cy5-labeled 20-DOI was topically administered to STZ-treated mice either as free 20-DOI or as TP/20-DOI. The fluorescence intensity of Cy5-labeled 20-DOI on the ocular surface was then monitored in the two groups at 0, 3, 6, and 9 h after instillation using an in vivo imaging system Bruker MI SE. b) Fluorescence intensity at each time point was quantified and compared between the two groups using Bruker MI SE (n = 5). Data are presented as mean ± SD. Statistical differences were analyzed by unpaired Student’s t-test. ns: *p* > 0.05; **: *p* < 0.01; ***: *p* < 0.001. STZ, streptozotocin; 20-DOI, 20-Deoxyingenol; TP/20-DOI, TSPBA-PVA loaded 20-Deoxyingenol; SD, standard deviation; ns, no significance.

**6 Analysis of Epithelial Junctional Integrity**

Zonula occludens-1 (ZO-1) staining was used to assess epithelial junctional integrity in murine corneas. At 24 h after corneal epithelial injury, both 20-DOI and TP/20-DOI microspheres increased ZO-1-positive junctional length in STZ mice, with a greater effect observed in the TP/20-DOI microsphere group (**Figure S6a**, **b**), suggesting enhanced restoration of epithelial barrier-associated junctional structure. Furthermore, TP/20-DOI increased ZO-1-positive junctional length, whereas this effect was reversed by co-administration of spautin-1 (**Figure S6c**, **d**), further indicating that autophagy contributes to epithelial reconstruction and recovery of junctional barrier integrity in TP/20-DOI microsphere-treated mice.

**Figure S6**

ZO-1 staining was performed to assess epithelial junctional integrity. a) Representative images of corneal ZO-1 staining in the Control, STZ, STZ + 20-DOI, STZ + TP, and STZ + TP/20-DOI groups, acquired with a 40× objective. b) Quantification of ZO-1-positive junctional length in the indicated groups (n = 4). c) Representative images of corneal ZO-1 staining in the Control, STZ, STZ + TP/20-DOI, and STZ + TP/20-DOI + SP groups, acquired with a 40× objective. d) Quantification of ZO-1-positive junctional length in the indicated groups (n = 4). Data are presented as mean ± SD. Statistical differences were analyzed by one-way ANOVA followed by Tukey's multiple comparisons test. ns: *p* > 0.05; *: *p* < 0.05; **: *p* < 0.01; ***: *p* < 0.001. ZO-1, zonula occludens-1; STZ, streptozotocin; 20-DOI, 20-Deoxyingenol; TP, TSPBA-PVA; TP/20-DOI, TSPBA-PVA loaded 20-Deoxyingenol; IF, immunofluorescence; SP, spautin-1; SD, standard deviation; ns, no significance.

**7 Corneal Transparency and Fibrosis Assessment**

Corneal transparency was quantitatively evaluated on day 14 after epithelial injury by measuring light transmittance using a microplate reader-based assay in the Control, STZ, STZ + 20-DOI, STZ + TP, and STZ + TP/20-DOI groups. No significant differences in corneal transparency were observed among these groups (**Figure S7a**). Furthermore, fibrosis was assessed by α-SMA immunofluorescence staining on days 7 and 14 after injury. No α-SMA-positive staining was detected in any group at either time point (**Figure S7b**, **c**), suggesting that overt fibrosis did not occur within the experimental observation period.

**Figure S7**

Assessment of corneal transparency and fibrosis. a) Quantification of corneal light transmittance in the Control, STZ, STZ + 20-DOI, STZ + TP, and STZ + TP/20-DOI groups (n = 4). b) Representative corneal immunofluorescence images showing DAPI and α-SMA staining in the Control and STZ groups at 7 days after epithelial abrasion, imaged with a 40× objective. c) Representative corneal immunofluorescence images showing DAPI and α-SMA staining in the Control and STZ groups at 14 days after epithelial abrasion, imaged with a 40× objective. Data are presented as mean ± SD. Statistical differences were analyzed by one-way ANOVA followed by Tukey's multiple comparisons test. ns: *p* > 0.05. DAPI, 4',6-diamidino-2-phenylindole; α-SMA, alpha-smooth muscle actin; STZ, streptozotocin; 20-DOI, 20-Deoxyingenol; TP, TSPBA-PVA; TP/20-DOI, TSPBA-PVA loaded 20-Deoxyingenol; SD, standard deviation; ns, no significance.

**8 TP/20-DOI Microspheres Show Comparable Efficacy to bFGF**

Basic fibroblast growth factor (bFGF) was used as a reference treatment for comparison with TP/20-DOI microspheres. TP/20-DOI microspheres exhibited greater therapeutic efficacy than bFGF in promoting corneal wound healing; however, no significant difference was observed between the two groups in terms of nerve repair (**Figure S8**).

**Figure S8**

Comparison of TP/20-DOI microspheres and bFGF in corneal wound healing in STZ-treated mice. a) Representative sodium fluorescein staining images of the corneal wound area in the Control, STZ, STZ + bFGF, and STZ + TP/20-DOI groups. b) Quantification of corneal wound closure in STZ-treated mice following TP/20-DOI or bFGF treatment (n = 4-6). c) Representative β-III-tubulin immunostaining images of corneas at 4 days after epithelial abrasion. d) Quantification of nerve density in the central cornea (1 mm × 1 mm) among the indicated groups (n = 6). e) Quantitative comparison of corneal sensitivity among the indicated groups at 1, 2, 3, and 4 days after epithelial abrasion. Data are presented as mean ± SD. Statistical differences were analyzed by one-way ANOVA followed by Tukey's multiple comparisons test. ns: *p* > 0.05, *: *p* < 0.05; **: *p* < 0.01, ***: *p* < 0.001. STZ, streptozotocin; bFGF, basic fibroblast growth factor; TP/20-DOI, TSPBA-PVA loaded 20-Deoxyingenol; SD, standard deviation; ns, no significance.

**9 Scratch Assay of HCECs**

To investigate whether 20-DOI or TP/20-DOI microspheres directly influence corneal epithelial wound closure, a scratch assay was conducted in human corneal epithelial cells (HCECs) under high-glucose (HG) conditions. Four groups were evaluated: HG, HG + 20-DOI, HG + TP, and HG + TP/20-DOI. Quantitative analysis of wound closure at 12 and 24 h showed no statistically significant differences among the groups (**Figure S9**), suggesting that neither free 20-DOI nor TP/20-DOI microspheres directly enhance epithelial wound closure.

**Figure S9**

Effects of 20-DOI, TP, and TP/20-DOI on wound closure of human corneal epithelial cells (HCECs) under high-glucose (HG) conditions after scratching (n = 3). Data are presented as mean ± SD. Statistical differences were analyzed by one-way ANOVA followed by Tukey's multiple comparisons test. ns: *p* > 0.05. 20-DOI, 20-Deoxyingenol; TP, TSPBA-PVA; TP/20-DOI, TSPBA-PVA loaded 20-Deoxyingenol; SD, standard deviation; ns, no significance.

**10 Evaluation of TNF-α-Mediated NF-κB Activation in the Regulation of CXCL3/5 Expression in Macrophages**

We next investigated whether NF-κB signaling was involved in the TP/20-DOI-induced suppression of CXCL3 and CXCL5 in RAW264.7 cells. TP/20-DOI treatment significantly reduced the p-p65/total p65 ratio compared with the HG group, indicating inhibition of NF-κB activation. Notably, the addition of TNF-α significantly restored the p-p65/total p65 ratio (**Figure S10a**). Consistent with these changes in NF-κB activity, TP/20-DOI microspheres significantly decreased the secretion of CXCL3 and CXCL5, whereas TNF-α treatment reversed these effects (**Figure S10b**). Together, these functional rescue results suggest that inhibition of NF-κB signaling contributes to the TP/20-DOI-mediated suppression of CXCL3 and CXCL5 expression.

**Figure S10**

Effects of TNF-α on TP/20-DOI-mediated NF-κB activation and CXCL3/5 expression in RAW264.7 cells. a) Ratio of phosphorylated NF-κB p65 to total p65 in RAW264.7 cell lysates from the HG, HG + TP/20-DOI, and HG + TP/20-DOI + TNF-α groups (n = 3). b) Concentrations of CXCL3 and CXCL5 in the corresponding culture supernatants, as determined by ELISA (n =3). Data are presented as mean ± SD. Statistical differences were analyzed by one-way ANOVA followed by Tukey's multiple comparisons test. ***: *p* < 0.001. HG, high glucose; TP/20-DOI, TSPBA-PVA loaded 20-Deoxyingenol.

**11 Effect of Spautin-1-Treated BMDMs on TP/20-DOI-Mediated Corneal Wound Repair in Diabetic Mice**

To further evaluate whether macrophage autophagy contributes to TP/20-DOI-mediated corneal epithelial repair, F4/80^+^ bone marrow-derived macrophages (BMDMs) from diabetic mice were isolated and divided into two groups. One group was treated with Spautin-1 for 3 hours to inhibit autophagy, while the other group received vehicle treatment. These cells were then injected into diabetic recipient mice via the tail vein at 0 and 12 hours after corneal epithelial injury, with 1 × 10^6^ cells administered per mouse at each time point. Corneal epithelial wound healing was monitored during the repair process, and corneas were harvested at 24 hours post-injury for whole-mount analysis of proliferating epithelial cells. The results showed that mice receiving Spautin-1-treated BMDMs exhibited significantly delayed corneal wound closure and markedly fewer proliferating epithelial cells compared with mice receiving vehicle-treated BMDMs (**Figure S11**). These findings further support that macrophage autophagy is required for the pro-repair effect of TP/20-DOI microspheres in diabetic corneal wound healing.

**Figure S11**

Evaluation of the effects of SP-treated BMDMs on TP/20-DOI-mediated corneal wound healing in diabetic mice. a) Sodium fluorescein staining of the corneal wound area. b) Comparison of corneal wound closure between STZ mice receiving vehicle-treated BMDMs plus TP/20-DOI eye drops and STZ mice receiving SP-treated BMDMs plus TP/20-DOI eye drops (n = 4-6). c) DAPI staining of corneal epithelial basal cells observed under a 40× objective lens. d) Comparison of the number of dividing epithelial basal cells at 24 hours after epithelial abrasion between the two groups (n = 4-6). Data are presented as mean ± SD. Statistical differences were analyzed by unpaired Student’s t-test. *: *p* < 0.05; **: *p* < 0.01, ***: *p* < 0.001. STZ, streptozotocin; BMDM, bone marrow-derived macrophage; SP: Spautin-1; TP/20-DOI, TSPBA-PVA loaded 20-Deoxyingenol.

**12 Cell Enumeration**

The cornea was partitioned into five distinct zones for analysis: Zone 1 (limbus), Zone 2 (peripheral cornea), and Zones 3–5 (**Figure S4**), with Zones 3–5 corresponding to the epithelial abrasion site. To evaluate epithelial proliferation and CXCL3/5 expression, a total of 17 microscopic fields spanning the entire cornea from limbus to limbus were imaged using a 40× objective. For neutrophil quantification, four quadrants within Zone 3 of the cornea were examined. One field of view was randomly selected per quadrant, and neutrophils present in each field were counted. The average neutrophil count across the four fields was used to represent the degree of neutrophil recruitment to the wound area.

**Figure S12**

A schematic view for measuring the expression of CXCL3/5 and counting the number of dividing epithelial cells and neutrophils.

**Table S1**. Actual drug-loading (DL) content and encapsulation efficiency (EE) of TP/20-DOI microspheres.

| Group | DL (%) | EE (%) |
| --- | --- | --- |
| TP/20-DOI 0.5 | 6.9 ± 1.3 | 84.9 ± 1.2 |
| TP/20-DOI 1 | 7.3 ± 0.8 | 86.6 ± 0.7 |
| TP/20-DOI 1.5 | 7.6 ± 1.1 | 87.4 ± 0.9 |

**Table S2**. Mucoadhesion and wash-off resistance of TP/20-DOI microspheres.

| Group | Work of adhesion (mN.mm) | Wash-off resistance (h) |
| --- | --- | --- |
| TP/20-DOI 0.5 | 40.37 ± 5.69 | 5.43 ± 0.32 |
| TP/20-DOI 1 | 42.12 ± 2.85 | 5.35 ± 0.27 |
| TP/20-DOI 1.5 | 44.33 ± 3.67 | 5.89 ± 0.38 |

**Table S3.** Primer sequences used for RT-qPCR

| Gene Name | Primer Sequence（5’～3’) | |
| --- | --- | --- |
| *Gapdh* | Forward | 5’-CAAGGACACTGAGCAAGAG-3’ |
|  | Reverse | 5’-TGCAGCGAACTTTATTGATG-3’ |
| *Map1lc3b* | Forward | 5’-ACCAAGATCCCAGTGATTAT-3’ |
|  | Reverse | 5’-CTCTCACTCTCGTACACTTC-3’ |
| *Atg14* | Forward | 5’-TTCAAAGCTTACACTGGACA-3’ |
|  | Reverse | 5’-CTATCGCAACTACACAGACA-3’ |
| *Atg10* | Forward | 5’-CCAGGTCAGGGCGAG-3’ |
|  | Reverse | 5’-TGCTGTGAATGTCTGATGAA-3’ |
| *Tnf* | Forward | 5’-CATCCCCAGAAAAGAC-3’ |
|  | Reverse | 5’-AGAAGATGATTGAGTGTGAG-3’ |
| *Il-1β* | Forward | 5'-GAAATGCCACCTTTTGACAG -3' |
|  | Reverse | 5'-TGGATGCTCTCATCAGGACA-3' |
| *Cd86* | Forward | 5’-GACCCCAGATGCACCAT-3’ |
|  | Reverse | 5’-GCAGTCCCATTGAAATAAGC-3’ |
| *Nos2* | Forward | 5’-CCCAGCCAGCCCAAC -3’ |
|  | Reverse | 5’-TGTCATGCAAAATCTCTCCA-3’ |
| *Cxcl3* | Forward | 5’-AAATCAGAGAAAAGCGATCC-3’ |
|  | Reverse | 5’-AGATGCAATTATACCCGTAGT-3’ |
| *Cxcl5* | Forward | 5’-GTTTGCTTAACCGTAACTCC-3’ |
|  | Reverse | 5’-TAGCTATGACTTCCACCGTA-3’ |
| *Mrc1* | Forward | 5'-CCGACATGCCAGGACGAAAG-3' |
|  | Reverse | 5'-AGGAGTTGTTGTGGGCTCTG-3' |
| *Arg1* | Forward | 5'-CTCCAAGCCAAAGTCCTTAGAG-3' |
|  | Reverse | 5'-AGGAGCTGTCATTAGGGACATC-3' |
| *Tgf-β* | Forward | 5′-ACCAACACACCCCATCTATGAG-3′ |
|  | Reverse | 5′-GGGGGTCAGCACCGGTAC-3′ |
| *Ccl22* | Forward | 5′-GGCACCTATCCAGTGCCACA-3′ |
|  | Reverse | 5′-TGGTGGACCAGCCTGAAACTC-3′ |
| *α-Kgdh* | Forward | 5'-ACATGAGGCCAGATAGGGGA-3' |
|  | Reverse | 5'-TTGGATTACACCGGCCTCAG-3' |
| *Idh* | Forward | 5'-GGAGAAGCCGGTAGTGGAGAT-3' |
|  | Reverse | 5'-GGTCTGGTCACGGTTTGGAA-3' |
